# Prospective directional associations between lipid measures and digital volume pulse-derived arterial stiffness parameters: evidence from cross-lagged panel analyses in the UK Biobank

**DOI:** 10.3389/fnut.2026.1942971

**Published:** 2026-10-29

**Authors:** Zihang Li, Bangwei Chen, Zehua Wu, Yaxin Li, Shengyin Zeng, Yueqi Lu, Xin Jin, Shida Zhu, Jianguo Zhang, Tao Li, Ying Shan

**Affiliations:** 1 Clinical Research Academy, Peking University Shenzhen Hospital, Peking University, Shenzhen, Guangdong Province, China; 2 BGI Genomics, Shenzhen, China; 3 Department of Chemical and Biomolecular Engineering, New York University, Brooklyn, NY, United States; 4 Department of Geriatrics, Tongji Hospital, Tongji Medical College, Huazhong University of Science and Technology, Wuhan, Hubei, China; 5 Frontier Science Center for Immunology and Metabolism, Medical Research Institute, Wuhan University, Wuhan, Hubei, China; 6 College of Life Sciences, University of Chinese Academy of Sciences, Beijing, China; 7 BGI Research, Shenzhen, China

**Keywords:** arterial stiffness, cardiovascular disease, cross-lagged panel model, dyslipidemia, lipid metabolism

## Abstract

**Background:**

Cardiovascular disease (CVD) is the leading cause of morbidity and mortality worldwide. Arterial stiffness and dyslipidemia are considered risk factors for CVD. However, no relevant prospective inference studies have been reported in the middle-aged and elderly populations. This study aimed to investigate the prospective directional associations between dyslipidemia and arterial stiffness.

**Methods:**

The study included 20,344 participants from the UK Biobank with available first follow-up data. After applying the inclusion and exclusion criteria, 2,989 participants remained, with a median follow-up interval of 3.0 years (interquartile range, 2.8–3.2). Prospective directional associations between arterial stiffness and dyslipidemia were confirmed by the classical cross-lagged panel model and three improved nonlinear cross-lagged panel models.

**Results:**

The baseline age of the participants was 58.7 ± 7.2 years, of which 51.7% were male. In males, cross-lagged analyses revealed a prospective directional association from apolipoprotein B (ApoB) to pulse wave reflection index (PWRI; False discovery rate-corrected, FDR *p* = 0.038). In females, triglyceride (TG) levels showed prospective directional associations with subsequent digital volume pulse-derived arterial stiffness index, PWRI, and pulse wave peak-to-peak time, as well as pulse rate (FDR *p* < 0.05). Sex differences were observed in the associations of ApoB and TG with subsequent PWRI (*p* < 0.05). However, after incorporating SBP as a third endogenous variable, these associations were no longer statistically significant (FDR *p* > 0.05).

**Conclusion:**

The results of this study suggest that dyslipidemia is prospectively associated with arterial stiffness, with predominantly directional associations from lipid measures to arterial stiffness parameters. However, the associations were no longer statistically significant in the sensitivity analysis incorporating SBP, suggesting that the observed associations may be sensitive to SBP.

## Introduction

Arterial stiffness is considered an independent predictor of cardiovascular disease (CVD), robustly predicting cardiovascular morbidity and mortality (1–3). Arterial stiffness refers to the decrease in arterial elasticity due to aging-induced fibrosis of the arterial intima. Elevated arterial stiffness leads to increased blood pressure (BP) and promotes vascular remodeling leading to atherosclerosis. Recognized risk factors of arterial stiffness include age, gender, smoking, BP, obesity, diabetes, and so on (4–7).

Dyslipidemia is a metabolic abnormality that is often recognized as a possible risk factor for arterial stiffness (8). Dyslipidemia is characterized by (1) high plasma triglyceride (TG) levels, (2) low high-density lipoprotein (HDL) levels and (3) high low-density lipoprotein (LDL) levels. These characteristics can combine with endothelial dysfunction to cause the formation of atherosclerotic plaques, which can lead to arterial stiffness (9). Previous studies showed that total cholesterol (TC), high-density lipoprotein cholesterol (HDL-C) (8), triglyceride-glucose (TyG) index (10), apolipoprotein B (ApoB), and non-HDL-C (11) are significantly associated with arterial stiffness.

Likewise, the prospective association between dyslipidemia and arterial stiffness is of high interest. Agbaje’s group conducted the first analysis of the causal association between the two by using a cross-lagged panel model (4, 5). They demonstrated that higher carotid-femoral pulse wave velocity (cfPWV) precedes low HDL and obesity in British adolescent (ages from 17 to 24). Cross-lagged panel model can mine prospective directional association between variables by predicting how much an individual will deviate from the group mean at a previous time point by another variable at the next time point (12). The study by Jia’s group demonstrated that elevated ApoB or non-HDL-C is a better predictor of arterial stiffness risk in the middle-aged and elderly Chinese population (11). Despite extensive research on the association between dyslipidemia and arterial stiffness, there is a significant gap in studies examining their prospective directional association, particularly in the middle-aged and elderly population.

To fill this knowledge gap, we investigated the prospective directional association between dyslipidemia and arterial stiffness using cross-lagged panel models in the middle-aged and elderly populations. Moreover, we developed nonlinear cross-lagged panel models to uncover latent associations.

## Methods

### Data availability

The UK Biobank is a prospective study that enrolled over 500,000 participants aged 40–69 between 2006 and 2010. The UK Biobank protocol received approval from the Northwest Multicenter Research Ethics Committee, and all participants provided written informed consent. The study collected comprehensive questionnaire data, physical measurements, and biological samples from the participants. The corresponding UK Biobank field identifiers, instances, and arrays for all variables used in the analyses are provided in Supplementary Table S1.

### Study cohort

The data utilized in this study were retrieved from the UK Biobank public repository. The UK Biobank comprises 502,462 participants aged between 40 and 69 years. A total of 20,344 participants had available first follow-up data and were therefore considered for the present analysis. Participants with incomplete digital volume pulse (DVP)-derived arterial stiffness parameters at baseline or follow-up were first excluded (*N* = 15,676), leaving 4,668 participants. Of these, 1,679 participants were further excluded because of incomplete lipid measurements at baseline or follow-up (Figure 1). Ultimately, 2,989 participants were included in the study to investigate the prospective directional association between dyslipidemia and arterial stiffness.

**Figure 1:**
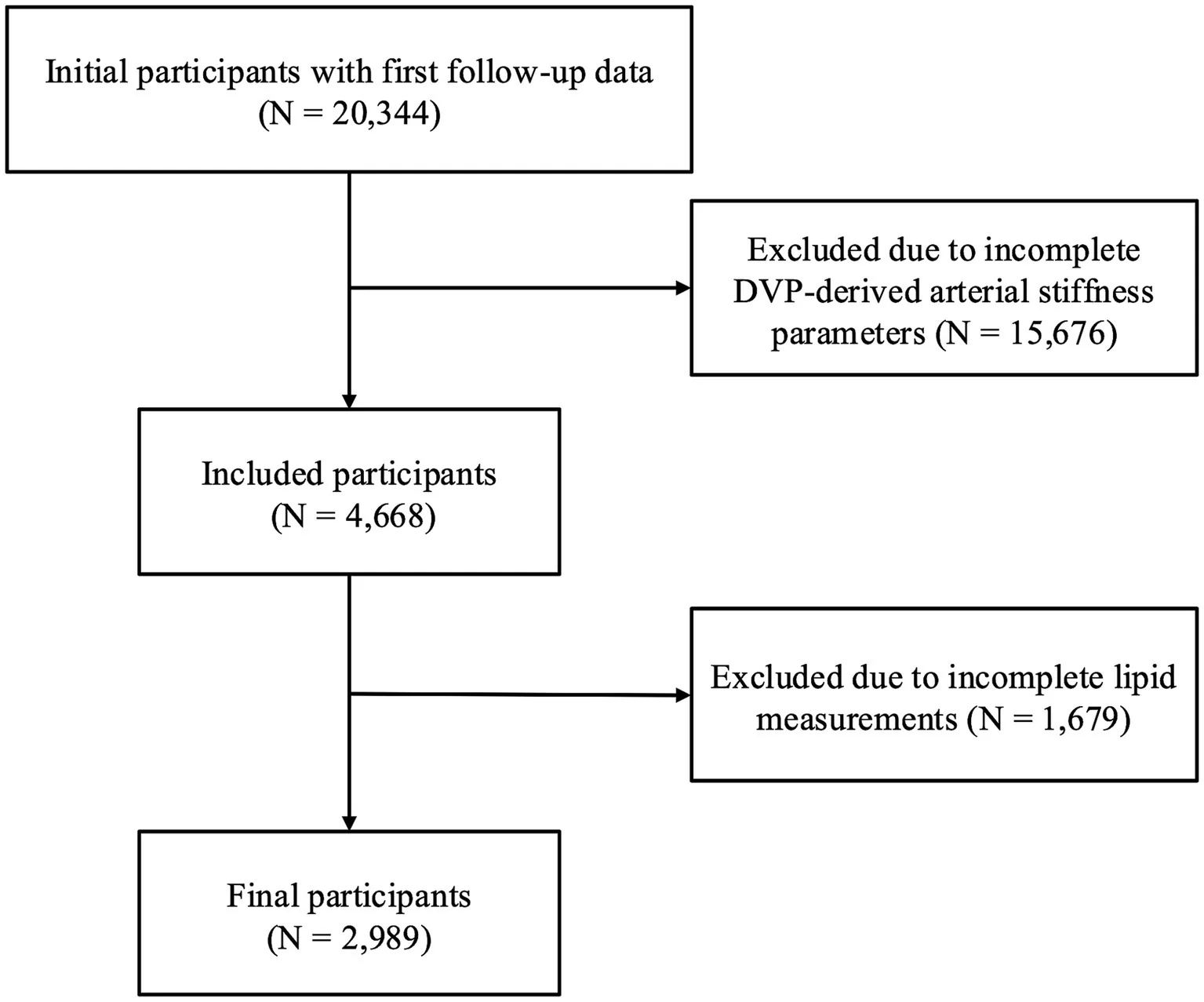
Flowchart of study participants.

### Anthropometric and laboratory data

Information about the participants’ demographics, lifestyle, and medical history was collected through touchscreen questionnaire (UKB Category 100,025). The height, weight, and systolic blood pressure (SBP) of these participants were measured by trained medical professionals. Body mass index (BMI) was calculated as weight in kilograms divided by the square of height in meters. Participants were defined as diabetic by self-reporting a history of diabetes at the time of questionnaire participation. Hypertension was defined as SBP ≥ 140 mmHg and/or diastolic blood pressure (DBP) ≥ 90 mmHg. Smoking status was defined as three conditions: “Never,” “Previous,” and “Current.”

### DVP-derived arterial stiffness parameters

The contour of the DVP may be used to estimate arterial stiffness (13). In this study, DVP-derived parameters, including arterial stiffness index (ASI), pulse wave reflection index (PWRI), pulse wave peak-to-peak time (PWPP), and pulse rate (PR), were obtained from pulse waveforms using PulseTrace PCA2 (CareFusion, United States). ASI was prespecified as the primary arterial stiffness endpoint and was obtained as the participant’s height divided by the PWPP. PWRI and PWPP were considered secondary DVP-derived parameters. PR was considered a pulse-related parameter rather than a direct measure of arterial stiffness.

### Lipid measurements

A series of biological samples were collected from participants and then analyzed on various automated systems to collect blood biochemical data. Lipid levels were assessed by TC, HDL-C, low-density lipoprotein cholesterol (LDL-C), TG, apolipoprotein A (ApoA), ApoB, and lipoprotein A (LpA). An AU5800 (Beckman Coulter, United States) was used to measure albumin, alkaline phosphatase (ALP), alanine aminotransferase (ALT), ApoA, ApoB, aspartate aminotransferase (AST), urea, calcium, cholesterol, c-reactive protein (CRP), gamma-glutamyl transferase (GGT), glucose, LpA, phosphate, total bilirubin (TBIL) total protein (TP), TG and urate; A high-throughput NMR-based metabolic biomarker profiling platform (Nightingale Health Ltd., Finland) was used to measure TC, HDL-C, and LDL-C; ADVIA 1800 (Siemens, United States) was used to measure cystatin c (CysC); Ltd. LIASON XL (DiaSorin, Italy) was used to measure IGF-1 and Vitamin D (VitD); Unicel Dxl 800 (Beckman Coulter, United States) was used to measure sex hormone binding globulin (SHBG) and testosterone. All biochemistry and NMR metabolomics data were directly extracted from the officially published UK Biobank datasets, which had undergone quality control and calibration procedures.

### Statistical analysis

For baseline participant characteristics, non-normally distributed continuous variables are expressed as median (quartiles), normally distributed continuous variables are expressed as mean ± standard deviation (SD), and categorical variables are expressed as numbers and percentages (%). For continuous variable data sets, *t*-tests (for normally distributed continuous variables) and Wilcoxon rank sum tests (for non-normally distributed continuous variables) were used to test for differences between genders. For the categorical variables data sets, the chi-square test was used to test for differences between genders.

We first constructed a classical linear cross-lagged panel model (CLPM) to assess the prospective directional associations between DVP-derived arterial stiffness parameters and blood lipid measures. Building on this, we further developed three distinct forms of nonlinear cross-lagged panel models. Three distinct forms of nonlinear cross-lagged panel models were developed. The polynomial regression-based cross-lagged model (PRCM) replaces the general linear regression component of the structural equation with a polynomial regression. The generalized additive cross-lagged model (GACM) introduces generalized additivity modeling into the structural equation framework, which enables the incorporation of a smooth nonlinear function of the predictors that captures the nonlinear relationship between the predictor and response variables. The mixed generalized cross-lagged model (mixed-GACM) further extends the GACM by allowing for random effects, thereby accommodating both fixed and random variations in the data.

The cross-lagged panel model is essentially a special kind of structural equation, and our approach to improving the three models involved replacing the general linear regression model in the structural equation with a different nonlinear model while keeping the overall structure of the structural equation unchanged. Repeatedly measured variables at baseline and follow-up were used in all models. Restricted cubic spline (RCS) with 5 knots was used to analyze nonlinear associations before cross-lagged analysis. Due to their non-normal distributions, TG, LpA, PWPP, and PWRI were log-transformed for the main cross-lagged analyses and other sensitivity analyses. The GACM and mixed-GACM sensitivity analyses using untransformed variables were conducted on their original scales. The covariate-adjusted models were as follows: model 1 was adjusted for Glucose, BMI, estimated glomerular filtration rate (eGFR), CRP, and IGF-1 (these commonly associated markers were adjusted to rule out interference from other common conditions that may co-occur with dyslipidemia and arterial stiffness); and model 2 was additionally adjusted for age, smoking, diabetes, SBP, albumin, ALP, ALT, AST, GGT, phosphate, TBIL, TP, CysC, urea, urate, testosterone, SHBG, calcium, and VitD, excluding DVP-derived parameters (ASI, PWRI, PWPP, and PR) and lipid-related measures (ApoA, ApoB, HDL-C, LDL-C, LpA, TG, and TC). All adjusted covariates included in these models were based on baseline measurements.

For the present analysis, the classical linear CLPM was preselected as the primary analysis model to assess prospective directional associations. The GACM was additionally fitted as an exploratory model to allow for potential nonlinear relationships without imposing a specific parametric functional form *a priori*. PRCM and mixed-GACM were used as alternative nonlinear models in sensitivity analyses. To assess the relative fit of the four models, we calculated the Akaike information criterion (AIC) and Bayesian information criterion (BIC). Model-fit comparisons were used to characterize the relative performance of the four models and were not used to select models based on the statistical significance of individual associations.

To assess whether the directional cross-lagged associations differed between males and females, we additionally conducted multi-group analyses by sex to test between-sex differences in the corresponding cross-lagged paths. The corresponding cross-lagged paths were freely estimated for males and females, and equality constraints were imposed separately on each path. Between-sex differences were assessed using Wald tests. To assess potential age-related heterogeneity, age-stratified analyses were performed according to age (<50 and ≥50 years).

### Mediation analysis

To further explore potential pathways underlying the directional cross-lagged association identified in the primary analysis, exploratory mediation analyses were performed with CRP as a candidate mediator. The mediation analyses examined whether follow-up CRP mediated the directional cross-lagged association between baseline lipid measures and follow-up DVP derived parameters. Models were adjusted for baseline CRP, the corresponding baseline outcome, glucose, BMI, eGFR, and IGF-1. Indirect, direct, and total effects were estimated using 5,000 bootstrap resamples.

### Sensitivity analysis

To assess the robustness of the main findings, several were conducted. First, PRCM and mixed-GACM were used as alternative nonlinear model specifications. Second, the GACM and mixed-GACM models were repeated using PWRI and PWPP on their original scales without log transformation. TG and LpA were also analyzed on their original scales, with a Gamma distribution and log link specified when either variable was modeled as the outcome. Covariates were directly included in these models. Third, SBP was incorporated as a third endogenous variable within the GACM to account for its potential time-varying confounding effect.

All statistical analyses were performed using R-4.2.1 (R Foundation for Statistical Computing, Vienna, Austria). The R package *mgcv* (version 1.9.1) was used to implement the GACM (14); package *gamm4* (version 0.2.7) was used to implement the mixed-GACM (15); and package *rms* (version 6.8.1) was used to implement the RCS (16). For multiple-testing correction, each lipid-DVP-derived parameter pair was treated as a separate e hypothesis family, with the two reciprocal cross-lagged directions included within the same family. The two reciprocal directions were grouped within the same family because they represent the two directional tests of the same pair of variables. Given the 7 lipid measures and 4 DVP-derived parameters examined, this resulted in 28 hypothesis families for each sex and each model. The Benjamini-Hochberg procedure was applied to control the false discovery rate (FDR) within each hypothesis family. Effect estimates (β) and their corresponding 95% confidence intervals (95% CI) were reported alongside raw and FDR-adjusted *p*-values. A two-sided value of *p* < 0.05 was considered to indicate statistical significance.

## Results

### Cohort study characteristics

The characteristics of the 2,989 eligible participants are shown in Table 1, with a median follow-up interval of 3 years (interquartile range, 2.8–3.2) and a mean age of 58.7 ± 7.2 years and 57.4 ± 7.0 years for males and females, respectively, of which 51.7% were male. Compared with females in the baseline data, males had higher SBP, smoking prevalence, prevalence of developing diabetes, ASI, PWRI, and TG (*p* < 0.001) and lower PR, PWPP, TC, HDL-C, LDL-C, and ApoA (*p* < 0 0.001). PWPP and PR (*p* < 0.001) were reduced in both male and female follow-up data compared with baseline data. PWRI (*p* < 0.001) increased in female follow-up data (Supplementary Table S2). Baseline characteristics were also compared between participants included in and excluded from the final analytical sample (Supplementary Table S3).

**Table 1:** Descriptive characteristics of cohort participants at baseline.

Characteristics	Male (*N* = 1,544)	Female (*N* = 1,445)	*p*-value
Age, years	58.7 ± 7.2	57.4 ± 7.0	<0.001
Diabetes, *n* (%)	86 (5.6)	38 (2.6)	<0.001
Smoking status, *n* (%)			
Never	830 (53.8)	965 (66.8)	<0.001
Previous	612 (39.6)	429 (29.7)	
Current	102 (6.6)	51 (3.5)	
SBP, mmHg	141.1 ± 16.8	136.2 ± 18.7	<0.001
BMI	27.2 ± 4.0	26.5 ± 4.9	<0.001
Follow-up time, years	3.0 (2.8–3.2)	3.0 (2.8–3.2)	0.659
Arterial stiffness			
ASI, m/s	10.1 (8.0–11.7)	8.6 (6.5–10.8)	<0.001
PWRI, %	75.0 (66.0–81.0)	67.0 (56.0–75.0)	<0.001
PWPP, ms	177.0 (149.0–221.0)	188.0 (152.0–254.0)	<0.001
PR, bpm	66.7 ± 11.9	68.8 ± 10.7	<0.001
log (PWPP)	5.2 ± 0.3	5.3 ± 0.3	<0.001
log (PWRI)	4.3 ± 0.2	4.1 ± 0.3	<0.001
Glucose profiles			
Glucose, mmol/L	5.0 (4.7–5.4)	5.0 (4.7–5.3)	0.017
Lipid profiles			
TC, mmol/L	5.5 ± 1.1	6.0 ± 1.1	<0.001
HDL-C, mmol/L	1.3 ± 0.3	1.6 ± 0.4	<0.001
LDL-C, mmol/L	3.5 ± 0.9	3.7 ± 0.9	<0.001
TG, mmol/L	1.6 (1.2–2.4)	1.3 (1.0–1.8)	<0.001
LpA, nmol/L	29.5 (10.7–55.6)	34.0 (12.4–55.9)	0.106
ApoA, g/L	1.5 ± 0.2	1.7 ± 0.3	<0.001
ApoB, g/L	1.0 ± 0.2	1.0 ± 0.2	0.010
log (TG)	0.5 ± 0.5	0.3 ± 0.5	<0.001
log (LpA)	3.3 ± 1.1	3.4 ± 1.0	0.112
Liver function-related indexes			
Albumin, g/L	45.6 ± 2.5	45.2 ± 2.6	<0.001
ALP, U/L	78.1 (66.2–91.4)	81.8 (67.9–98.3)	<0.001
ALT, U/L	23.2 (18.0–30.9)	17.6 (14.0–22.5)	<0.001
AST, U/L	26.2 (22.8–30.6)	23.2 (20.2–27.2)	<0.001
GGT, U/L	31.2 (22.7–45.5)	21.0 (15.9–30.7)	<0.001
Phosphate, mmol/L	1.1 ± 0.2	1.2 ± 0.1	<0.001
TBIL, umol/L	9.2 (7.6–11.7)	7.4 (6.1–9.2)	<0.001
TP, g/L	72.4 ± 3.9	72.6 ± 3.9	0.300
Kidney function-related indexes			
eGFR, mL/min/1.73 m^2^	92.5 ± 15.8	91.1 ± 16.1	0.017
CysC, mg/L	0.9 (0.8–1.0)	0.8 (0.8–0.9)	<0.001
Urea, U/L	5.4 (4.7–6.2)	5.1 (4.4–6.0)	<0.001
Urate, umol/L	345.4 (301.9–391.4)	255.8 (219.2–301.2)	<0.001
Hormones			
IGF-1, nmol/L	22.2 ± 5.2	21.3 ± 5.8	<0.001
Testosterone, nmol/L	12.1 (9.9–14.5)	1.1 (0.8–1.3)	<0.001
SHBG, nmol/L	39.8 (30.0–50.9)	57.2 (41.7–78.8)	<0.001
Inflammatory factors			
CRP, mg/L	1.1 (0.6–2.2)	1.2 (0.6–2.6)	0.141
Other biochemical indexes			
Calcium, mmol/L	2.4 ± 0.1	2.4 ± 0.1	<0.001
VitD, nmol/L	46.7 (33.2–60.0)	47.5 (33.5–60.6)	0.544

The values are mean (SD) and median (interquartile range) except for diabetes, smoking status in percentage. Log (PWRI), log (PWPP), log (TG) and log (LpA) are the base-e logarithm of PWRI, PWPP, TG and LpA, respectively. Higher ASI and PWRI values indicate greater arterial stiffness, whereas higher PWPP values indicate lesser arterial stiffness. For categorical variables: the *p*-value was calculated using the Fisher exact test; for normally distributed continuous variables: the *p*-value was calculated using a *t*-test; for non-normally distributed continuous variables, the *p*-value was calculated using the Wilcoxon rank sum test. ASI, arterial stiffness index; PWRI, pulse wave reflection index; PWPP, pulse wave peak to peak time; PR, pulse rate; SBP, systolic blood pressure; TC, cholesterol; HDL-C, high-density lipoprotein cholesterol; LDL-C, low-density lipoprotein cholesterol; TG, triglycerides; LpA, lipoprotein A; ApoA, apolipoprotein A; ApoB, apolipoprotein B; BMI, Body mass index; ALP, alkaline phosphatase; ALT, alanine aminotransferase; AST, aspartate aminotransferase; GGT, gamma-glutamyl transferase; TBIL, total bilirubin; TP, total protein, eGFR, estimated glomerular filtration rate; CysC, cystatin C; SHBG, sex hormone binding globulin; CRP, c-reactive protein; VitD, vitamin D.

### Model fit comparison

Model fit comparison across all cross-lagged models demonstrated that GACM achieved the lowest Mean AIC (7087.389), whereas linear CLPM showed the lowest Mean BIC (7111.757), with only small differences between the two (ΔAIC = 0.358; ΔBIC = 0.561) (Table 2). Given the small differences in model fit and the greater parsimony of the linear model, the linear CLPM was selected as the primary analysis model. The GACM was subsequently used as an exploratory analysis to investigate potential nonlinear prospective directional associations.

**Table 2:** Model fit comparison of the four cross-lagged panel model specifications.

Model	AIC (mean)	Δ AIC (mean)	BIC (mean)	Δ BIC (mean)
Baseline framework				
Linear CLPM	7087.747	0.358	7111.757	0.000
Nonlinear framework				
GACM	7087.389	0.000	7112.318	0.561
mixed-GACM	7091.473	4.084	7127.489	15.732
PRCM	7089.089	1.700	7125.105	13.348

AIC, Akaike information criterion; BIC, Bayesian information criterion; CLPM, cross-lagged panel model; GACM, generalized additive cross-lagged model; PRCM, polynomial regression cross-lagged model. Mixed-GACM, mixed generalized cross-lagged model. Lower AIC and BIC values indicate better relative model fit. ΔAIC and ΔBIC represent the difference from the model with the lowest corresponding information criterion within the same comparison.

### Directional cross-lagged association of blood lipid measures and arterial stiffness parameters

The results of Cross-lagged panel model 1, which included adjustments for the covariates Glucose, BMI, eGFR, CRP, and IGF-1, revealed a significant negative association between baseline ApoB and follow-up PWRI in males (β2 = −0.057; false discovery rate-corrected (FDR) *p* = 0.049; 95% CI, −0.106 to −0.007) (Figure 2A and Supplementary Table S4). Higher TG levels at baseline were associated with higher ASI (β2 = 0.071; FDR *p* = 0.013; 95% CI, 0.020–0.122) and lower PWPP (β2 = −0.066; FDR *p* = 0.023; 95% CI, −0.117 to −0.015) at follow-up in females (Figures 2B,C). We further analyzed the baseline arterial stiffness parameters and follow-up lipid measures to examine prospective associations in the reverse direction (FDR *p* > 0.1).

**Figure 2:**
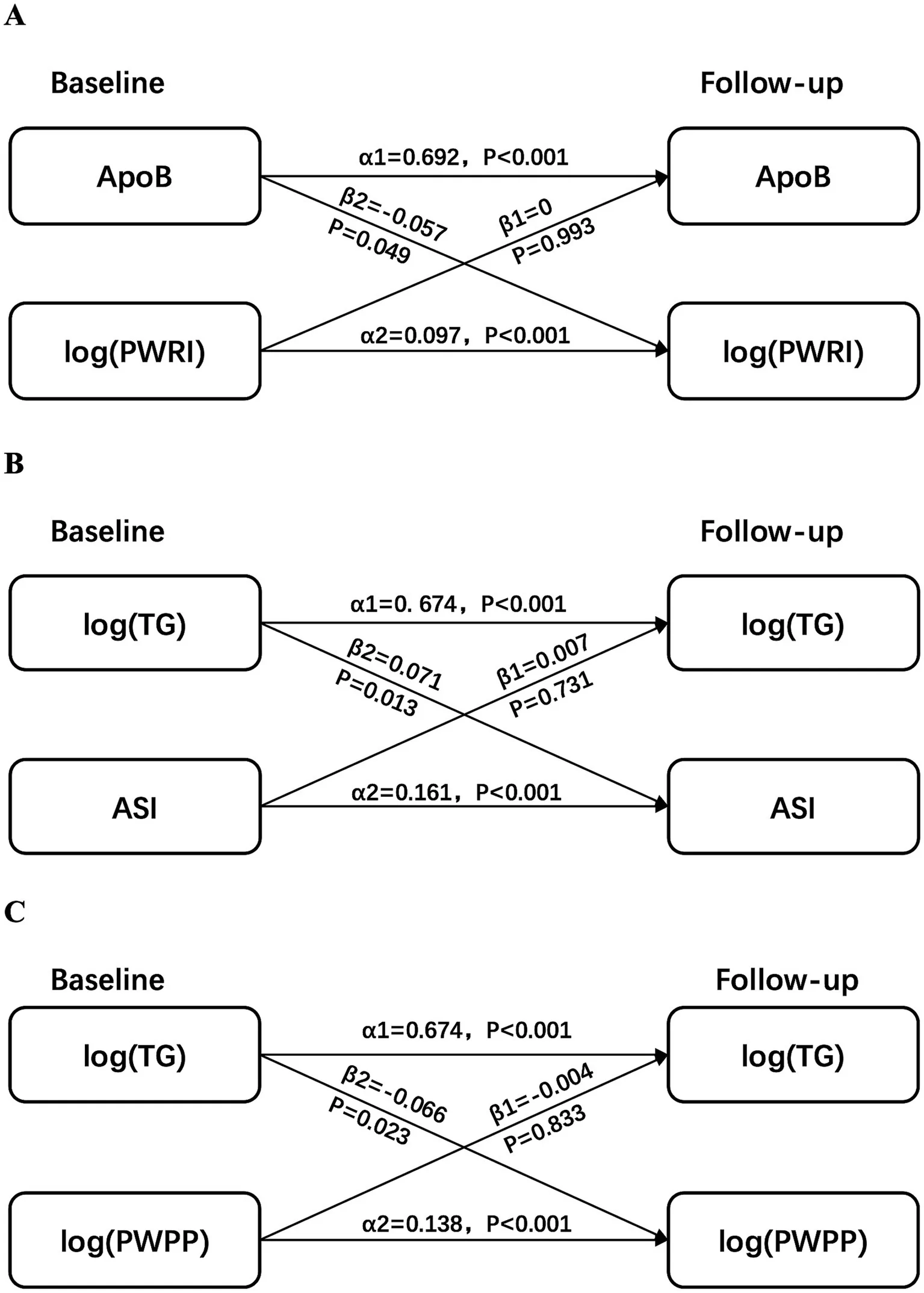
Directional cross-lagged associations between lipid measures and arterial stiffness parameters in model 1. **(A)** Directional cross-lagged association between ApoB and PWRI in males. **(B)** Directional cross-lagged association between TG and ASI in females. **(C)** Directional cross-lagged association between TG and PWPP in females. Analyses were adjusted for Age, Smoking, Diabetes, SBP, Glucose, eGFR, CRP, IGF1, and BMI at baseline. Benjamini-Hochberg FDR *p*-values were calculated for multiple tests. ASI, arterial stiffness index; PWRI, pulse wave reflection index; PWPP, pulse wave peak to peak time; TG, triglycerides; ApoB, apolipoprotein B.

The results of Cross-lagged panel model 2 revealed higher TG at baseline was associated with higher PWRI (β2 = 0.071; FDR *p* = 0.012; 95% CI, 0.020–0.122), but lower PR (β2 = −0.053; FDR *p* = 0.027; 95% CI, −0.094 to −0.011) at follow-up in females (Figures 3A,B and Supplementary Table S5). We further analyzed to examine prospective associations in the reverse direction (FDR *p* > 0.1).

**Figure 3:**
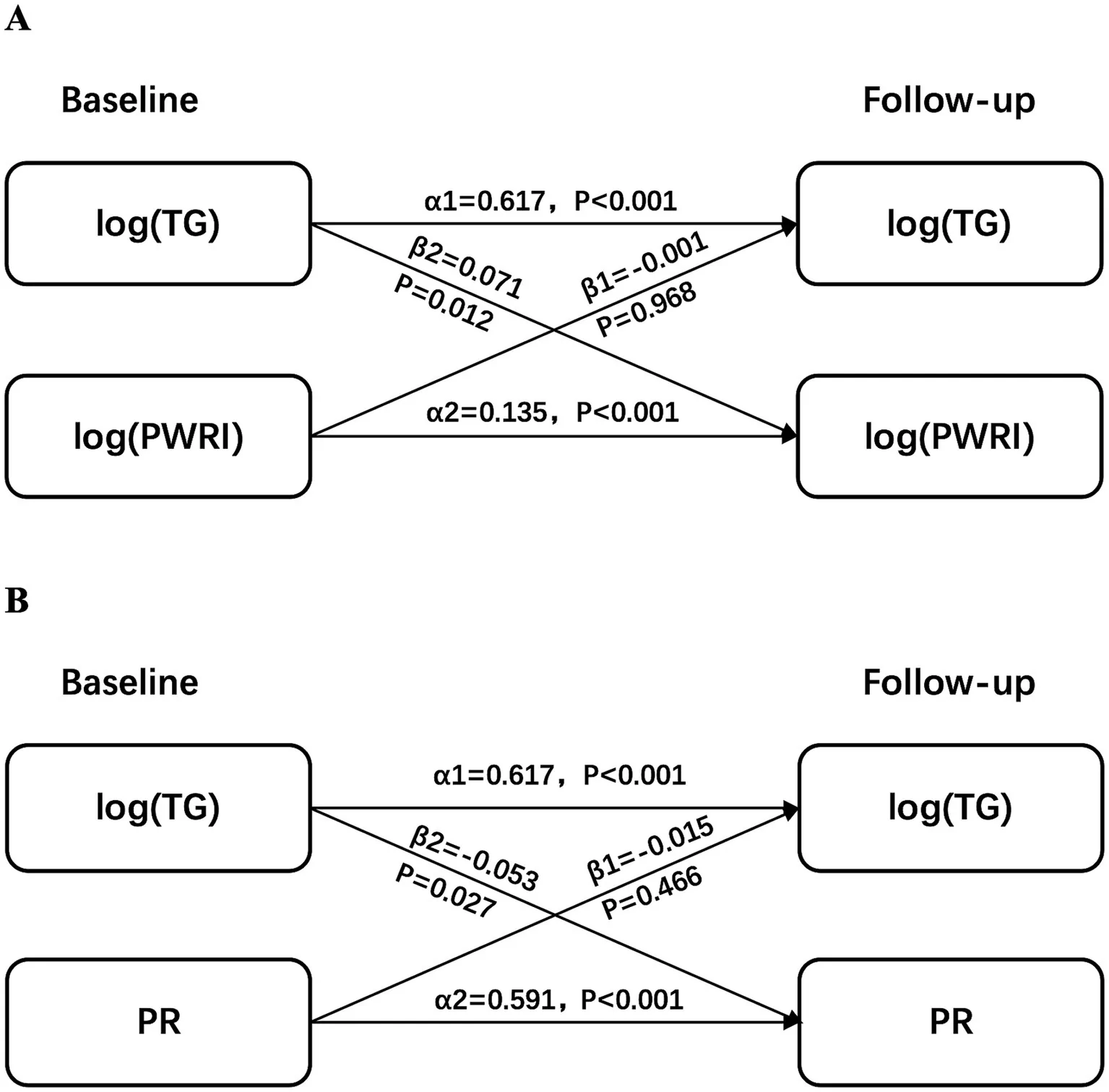
Directional Cross-Lagged Associations Between Lipid Measures and Arterial Stiffness Parameters in Model 2. **(A)** Directional cross-lagged association between TG and PWRI in females. **(B)** Directional cross-lagged association between TG and PR in females. Analyses were adjusted for Age, Smoking, Diabetes, SBP, Glucose, eGFR, CRP, IGF1, BMI, Albumin, ALP, ALT, AST, GGT, Phosphate, TBIL, TP, CysC, Urea, Urate, Testosterone, SHBG, Calcium and VitD at baseline. Benjamini-Hochberg FDR *p*-values were calculated for multiple tests. PWRI, pulse wave reflection index; PR, pulse rate; TG, triglycerides.

### Nonlinear directional cross-lagged association of blood lipid measures and arterial stiffness parameters

In males, by RCS, we observed a nonlinear association between PWPP and ApoA (nonlinear *p* = 0.031), ApoB (nonlinear *p* = 0.032), HDL-C (nonlinear *p* = 0.010), LDL-C (nonlinear *p* = 0.020), and TC (nonlinear *p* = 0.016) (Figure 4A). Specifically, the data revealed that as PWPP increased, ApoA exhibited a quadratic pattern. This was evidenced by the observation that as PWPP increased, ApoA initially decreased, reached a minimum, and then began to rise. In females, nonlinear associations were found between PWPP and ApoB (nonlinear *p* = 0.007), LDL-C (nonlinear *p* = 0.003), and TC (nonlinear *p* = 0.006) (Figure 4B). Specifically, the data revealed that ApoB exhibited a quadratic pattern with increasing PWPP. ApoB initially increased with PWPP, reached a maximum, and then started to decrease. These findings suggest that some associations between lipid measures and DVP-derived parameters may not follow a simple linear pattern. Therefore, we further applied nonlinear cross-lagged models to evaluate potential nonlinear directional cross-lagged associations between lipid measures and DVP-derived parameters.

**Figure 4:**
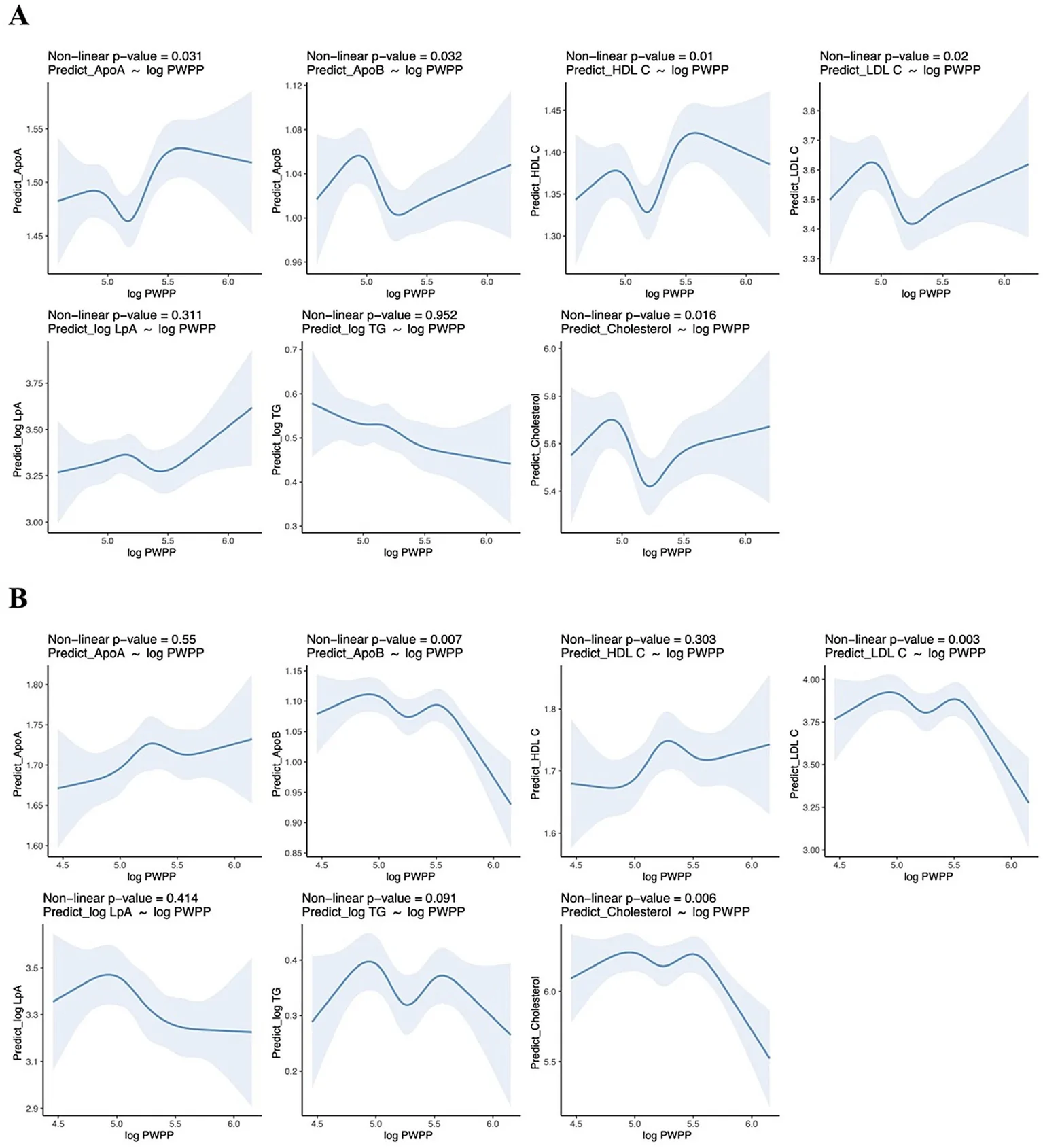
Nonlinear associations between lipid measures and arterial stiffness parameters in restricted cubic spline. **(A)** Nonlinear association between PWPP and ApoA, ApoB, HDL-C, LDL-C, LpA, TG, and Cholesterol in males. **(B)** Nonlinear association between PWPP and ApoA, ApoB, HDL-C, LDL-C, LpA, TG, and Cholesterol in females. PWPP, pulse wave peak to peak time; HDL-C, high-density lipoprotein cholesterol; LDL-C, low-density lipoprotein cholesterol; TG, triglycerides; LpA, lipoprotein A; ApoA, apolipoprotein A; ApoB, apolipoprotein B.

We next applied the GACM to characterize potentially nonlinear directional cross-lagged associations between lipid measures and DVP-derived parameters. In females, TG showed significant nonlinear associations with ASI (effective degrees of freedom (EDF) = 2.886; FDR *p* = 0.002) and PWPP (EDF = 2.885; FDR *p* = 0.003) (Table 3). The smooth function indicated that ASI initially decreased as TG increased, followed by an increase and a subsequent decline after reaching a peak, whereas PWPP initially increased, followed by a decline and a subsequent increase after reaching a trough (Figure 5A). In contrast, higher baseline TG was associated with higher PWRI (EDF = 1.000; FDR *p* = 0.012) and lower PR (EDF = 1.000; FDR *p* = 0.027) at follow-up, indicating essentially linear associations. In males, we did not observe significant nonlinear cross-lagged associations between TG and ASI, PWRI, PWPP, or PR (FDR *p* > 0.05). In addition, ApoB showed a significant nonlinear association with subsequent PWRI (EDF = 2.780; FDR *p* = 0.038). The smooth function indicated that PWRI initially decreased as ApoB increased, followed by a slight increase and a subsequent decline (Table 3; Figure 5B).

**Table 3:** Directional cross-lagged associations between lipid measures and arterial stiffness parameters in the generalized additive cross-lagged model.

Association	Male				Female			
	EDF	*F*	Raw *P*	FDR *P*	EDF	*F*	Raw *P*	FDR *P*
ASI → ApoA	1.000	3.316	0.069	0.138	1.266	0.555	0.412	0.412
ApoA → ASI	1.000	0.043	0.836	0.836	2.032	1.774	0.231	0.412
ASI → ApoB	1.000	0.007	0.931	0.931	1.000	1.469	0.226	0.226
ApoB → ASI	1.000	0.310	0.578	0.931	2.683	1.604	0.163	0.226
ASI → HDL-C	1.769	2.162	0.112	0.224	1.233	0.492	0.444	0.545
HDL-C → ASI	1.220	0.120	0.749	0.749	1.034	0.426	0.545	0.545
ASI → LDL-C	1.000	0.175	0.676	0.676	1.000	1.170	0.280	0.280
LDL-C → ASI	1.000	0.467	0.494	0.676	1.000	1.775	0.183	0.280
ASI → log(LpA)	1.000	0.227	0.633	0.633	1.668	0.914	0.379	0.508
log(LpA) → ASI	2.064	0.724	0.381	0.633	1.000	0.439	0.508	0.508
ASI → log(TG)	1.000	1.674	0.196	0.392	1.000	0.025	0.876	0.876
log(TG) → ASI	1.217	0.670	0.568	0.568	2.886	5.551	0.001	0.002
ASI → TC	1.000	0.012	0.913	0.913	1.000	0.589	0.443	0.443
TC → ASI	1.000	0.279	0.597	0.913	1.000	1.394	0.238	0.443
log(PWRI) → ApoA	1.000	2.198	0.138	0.217	1.000	0.092	0.761	0.761
ApoA → log(PWRI)	1.000	1.524	0.217	0.217	1.828	0.888	0.512	0.761
log(PWRI) → ApoB	2.682	1.008	0.309	0.309	1.000	0.223	0.637	0.637
ApoB → log(PWRI)	2.780	3.352	0.019	0.038	1.000	2.592	0.108	0.215
log(PWRI) → HDL-C	1.000	2.168	0.141	0.209	1.000	0.260	0.610	0.851
HDL-C → log(PWRI)	1.000	1.578	0.209	0.209	1.000	0.035	0.851	0.851
log(PWRI) → LDL-C	2.683	0.922	0.365	0.365	1.000	0.000	0.994	0.994
LDL-C → log(PWRI)	2.788	2.801	0.044	0.089	1.000	3.850	0.050	0.100
log(PWRI) → log(LpA)	1.000	0.505	0.477	0.477	1.000	0.430	0.512	0.512
log(LpA) → log(PWRI)	1.675	1.708	0.177	0.353	1.000	2.215	0.137	0.274
log(PWRI) → log(TG)	1.000	0.394	0.530	0.530	1.000	0.003	0.959	0.959
log(TG) → log(PWRI)	1.436	2.299	0.180	0.360	1.000	7.601	0.006	0.012
log(PWRI) → TC	1.000	0.038	0.845	0.845	1.000	0.066	0.797	0.797
TC → log(PWRI)	2.826	3.000	0.033	0.066	1.000	4.353	0.037	0.074
log(PWPP) → ApoA	1.000	2.693	0.101	0.202	1.000	1.490	0.222	0.296
ApoA → log(PWPP)	1.000	0.017	0.897	0.897	1.874	1.374	0.296	0.296
log(PWPP) → ApoB	1.000	0.078	0.780	0.780	2.526	1.691	0.120	0.120
ApoB → log(PWPP)	1.000	0.932	0.334	0.669	2.723	2.025	0.092	0.120
log(PWPP) → HDL-C	1.854	1.665	0.198	0.395	1.000	1.539	0.215	0.430
HDL-C → log(PWPP)	1.233	0.093	0.900	0.900	1.281	0.203	0.851	0.851
log(PWPP) → LDL-C	1.000	0.000	0.995	0.995	2.774	2.757	0.033	0.065
LDL-C → log(PWPP)	1.000	0.943	0.332	0.663	1.000	2.128	0.145	0.145
log(PWPP) → log(LpA)	1.000	0.350	0.554	0.554	1.000	1.060	0.303	0.387
log(LpA) → log(PWPP)	1.610	0.630	0.489	0.554	1.000	0.747	0.387	0.387
log(PWPP) → log(TG)	1.000	0.816	0.367	0.706	1.000	0.005	0.946	0.946
log(TG) → log(PWPP)	1.321	0.461	0.706	0.706	2.885	5.199	0.002	0.003
log(PWPP) → TC	1.000	0.161	0.688	0.688	2.782	2.480	0.047	0.094
TC → log(PWPP)	1.000	0.717	0.397	0.688	1.000	1.987	0.159	0.159
PR → ApoA	1.811	1.478	0.261	0.522	1.000	0.015	0.902	0.902
ApoA → PR	1.385	0.340	0.560	0.560	1.000	0.623	0.430	0.860
PR → ApoB	1.000	0.312	0.577	0.577	1.847	1.044	0.307	0.307
ApoB → PR	2.377	0.992	0.283	0.566	2.646	1.173	0.301	0.307
PR → HDL-C	2.681	2.392	0.121	0.241	1.000	0.298	0.585	0.799
HDL-C → PR	1.000	0.264	0.607	0.607	1.000	0.065	0.799	0.799
PR → LDL-C	2.312	0.956	0.532	0.532	1.849	1.424	0.249	0.249
LDL-C → PR	2.284	0.937	0.283	0.532	2.742	1.797	0.126	0.249
PR → log(LpA)	1.000	0.038	0.846	0.846	1.000	0.279	0.597	0.639
log(LpA) → PR	1.563	0.638	0.447	0.846	1.000	0.221	0.639	0.639
PR → log(TG)	1.000	0.322	0.571	0.654	2.637	1.191	0.300	0.300
log(TG) → PR	1.498	0.387	0.654	0.654	1.000	6.111	0.014	0.027
PR → TC	2.644	1.807	0.219	0.342	1.979	1.842	0.182	0.364
TC → PR	2.278	0.789	0.342	0.342	2.370	0.766	0.429	0.429

EDF indicates the effective degrees of freedom for the cross-lagged regression. Benjamini-Hochberg false discovery rate-corrected (FDR) *P*-value for multiple testing. Analyses were adjusted for Age, Smoking, Diabetes, SBP, Glucose, eGFR, CRP, IGF1, BMI, Albumin, ALP, ALT, AST, GGT, Phosphate, TBIL, TP, CysC, Urea, Urate, Testosterone, SHBG, Calcium and VitD at baseline. For categorical variables: the *p*-value was calculated using the Fisher exact test; for normally distributed continuous variables: the *p*-value was calculated using a *t*-test; for non-normally distributed continuous variables, the *p*-value was calculated using the Wilcoxon rank sum test. ASI, arterial stiffness index; PWRI, pulse wave reflection index; PWPP, pulse wave peak to peak time; PR, pulse rate; SBP, systolic blood pressure; TC, cholesterol; HDL-C, high-density lipoprotein cholesterol; LDL-C, low-density lipoprotein cholesterol; TG, triglycerides; LpA, lipoprotein A; ApoA, apolipoprotein A; ApoB, apolipoprotein B; BMI, Body mass index; ALP, alkaline phosphatase; ALT, alanine aminotransferase; AST, aspartate aminotransferase; GGT, gamma-glutamyltransferase; TBIL, total bilirubin; TP, total protein, eGFR, estimated glomerular filtration rate; CysC, cystatin C; SHBG, sex hormone binding globulin; CRP, c-reactive protein; VitD, vitamin D.

**Figure 5:**
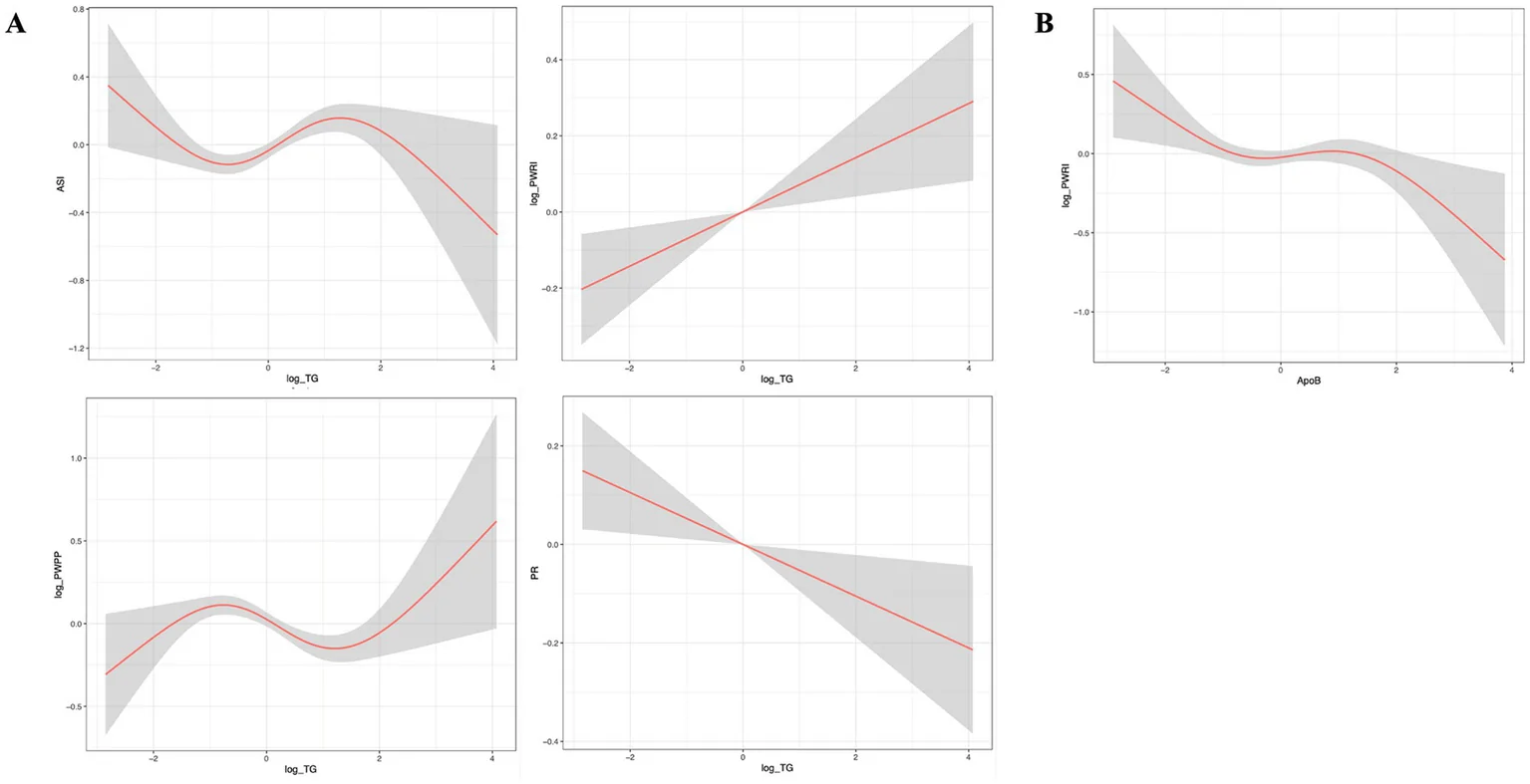
Smooth functions of directional cross-lagged associations between lipid measures and arterial stiffness parameters from GACM. **(A)** Smooth functions of directional cross-lagged associations in females. **(B)** Smooth functions of directional cross-lagged associations in males. Solid lines represent the estimated smooth functions, and shaded areas indicate 95% CI. An EDF of approximately 1 indicates an essentially linear association, whereas higher EDF values indicate nonlinear patterns. Only associations with FDR *p* < 0.05 are shown.

Formal between-sex comparisons were additionally conducted for the main directional cross-lagged associations identified in the primary CLPM and exploratory GACM analyses. Significant between-sex differences were observed for the paths from ApoB to subsequent PWRI (*p* = 0.020) and from TG to subsequent PWRI (*p* = 0.004), whereas the other main directional cross-lagged associations showed no significant between-sex differences (all *p* > 0.05) (Supplementary Table S6).

### Mediation analysis

In males, the indirect effect of ApoB on PWRI through CRP was not significant (*p* = 0.497). In females, CRP did not significantly mediate the associations of TG with ASI (*p* = 0.220), PWRI (*p* = 0.766), PWPP (*p* = 0.244), or PR (*p* = 0.162). However, the direct and total effects of TG on ASI remained significant (both *p* = 0.004) (Supplementary Table S7).

### Age-stratified analysis

Age-stratified analyses identified several significant directional cross-lagged associations among participants aged <50 years, including ApoB with PWRI and PWPP, TG with ASI, LDL-C with ASI and PWPP, and TC with PWPP (FDR *p* < 0.05). Associations of ASI and PWPP with ApoA were also significant (FDR *p* < 0.05). In contrast, no statistically significant associations were observed among participants aged ≥50 years (Supplementary Table S8). Formal interaction testing identified significant age differences for limited associations, including the association of TG with ASI, LDL-C with ASI, and TC with PWPP (FDR *p* < 0.05), whereas the remaining associations did not show significant age interactions (FDR *p* ≥ 0.05) (Supplementary Table S9).

### Sensitivity analysis

The mixed-GACM results were consistent with the findings from the primary CLPM and exploratory GACM analyses, with the same significant associations observed (Supplementary Table S10). In the PRCM results, we did not observe associations between DVP-derived parameters and lipid-related indices in males (all FDR *p* > 0.05) (Supplementary Table S11). In females, the quadratic and cubic terms of baseline TG were significantly associated with follow-up ASI (FDR *P_primary_* = 0.642, FDR *P_quadratic_* = 0.013, FDR *P_cubic_* = 0.013) and PWPP (FDR *P_primary_* = 0.477, FDR *P_quadratic_* = 0.041, FDR *P_cubic_* = 0.037), respectively, supporting nonlinear associations between TG and these parameters.

In sensitivity analyses using GACM and mixed-GACM with variables analyzed on their original scales, no directional cross-lagged associations remained statistically significant after FDR correction in females. In males, the association between ApoB and PWRI remained significant in both analyses (FDR *p* = 0.039 and 0.031) (Supplementary Table S12).

In an additional sensitivity analysis incorporating SBP as a third endogenous variable within the GACM, the previously directional cross-lagged associations were no longer significant (FDR *p* > 0.05). However, TG remained associated with subsequent PR (FDR *p* = 0.038), and PR was associated with subsequent TC in females (FDR *p* = 0.018) (Supplementary Table S13).

## Discussion

This study of 2,989 eligible participants resulted in a directional cross-lagged association between blood lipid measures and DVP-derived parameters. In the primary CLPM analysis, we identified several associations suggesting that lipid measures showed prospective directional associations with subsequent DVP-derived parameters. Importantly, the exploratory GACM analysis yielded consistent findings, higher baseline TG levels were prospectively associated with higher ASI at follow-up in females. TG was also associated with subsequent PWRI, PWPP, and PR in females, whereas baseline ApoB was associated with subsequent PWRI in males. Formal between-sex comparisons indicated significant sex differences in the directional cross-lagged associations of ApoB with PWRI and TG with PWRI, whereas the other main cross-lagged associations did not differ significantly between sexes. Sensitivity analyses using different model specifications and original-scale variables provided partial support for the robustness of the observed associations, although the statistical significance of individual associations varied across specifications. However, after incorporating SBP as a third endogenous variable, the previously directional cross-lagged associations were no longer statistically significant, suggesting these associations may be influenced by the dynamic changes in SBP. Overall, our findings suggest that prospective directional associations were predominantly observed from lipid measures to arterial stiffness parameters, rather than vice versa.

Several previous cohort studies have demonstrated that dyslipidemia is a determinant of arterial stiffness and that the risk of dyslipidemia is independently associated with arterial stiffness (17, 18). Moreover, several studies have demonstrated that some lipid markers, including TC, non-HDL-C, and TG, are positively associated with arterial stiffness, whereas HDL-C is negatively associated with arterial stiffness (7, 19). Increased arterial stiffness has been demonstrated as an early risk marker for developing type 2 diabetes, attributed to factors like endothelial dysfunction (17, 20). And dyslipidemia has been identified as a major driver of increased arterial stiffness in type 2 diabetes (21). Therefore, dyslipidemia is associated with and acts as a determinant of arterial stiffness in patients with type 2 diabetes. Furthermore, in hypertensive patients, dyslipidemia serves as a potential risk factor for arterial stiffness (18).

Agbaje et al. employed cross-lagged panel modeling to illustrate that higher blood glucose precedes higher arterial stiffness and that heightened arterial stiffness does not anticipate a heightened risk of dyslipidemia in British adolescents (4). In our present study, we observed that some lipid measures showed prospective directional associations with subsequent DVP-derived parameters. However, arterial stiffness did not emerge as a risk factor for dyslipidemia, consistent with the findings of the British adolescent study.

The current study revealed predominantly prospective directional associations from dyslipidemia to arterial stiffness. Dyslipidemia is characterized by elevated levels of LDL-C and TG, and reduced levels of HDL-C (22). Our study demonstrated that higher baseline ApoB levels were prospectively associated with subsequent PWRI in males, with a significant between-sex difference. This finding suggests a prospective association between ApoB and wave reflection. ApoB is the principal apolipoprotein on LDL particles and plays a crucial role in LDL-C assembly. The initial event in atherosclerosis formation involves the accumulation of LDL-C and ApoB in the arterial wall, leading to the development of atherosclerotic plaques, which subsequently cause stenosis and arterial stiffness (23). Moreover, elevated levels of ApoB have been demonstrated to predict the risk of arterial stiffness (11). PWRI has been shown to reflect the compliance of both large and small arteries and may therefore provide an indirect measure of arterial stiffness (24). Retention and accumulation of ApoB in the arterial wall can promote lipid accumulation and vascular remodeling, thereby affecting arterial compliance and vascular function. There are limited studies investigating the prospective association between ApoB and vascular changes, and our study contributes to filling this gap.

TG has been shown to be the standardized lipid with the strongest correlation with arterial stiffness and to predict the progression of arterial stiffness (23). The hydrolysis of TG by lipoprotein lipase generates numerous pro-inflammatory mediators, which can alter vascular endothelial function and promote plaque formation, ultimately contributing to arterial stiffness (25). Kim et al. reported a significant association between TG levels and arterial stiffness in both males and females (26), whereas Dabelea et al. found no significant association between TG levels and arterial stiffness (27). In our study, the GACM showed that TG levels were prospectively associated with all four DVP-derived parameters (ASI, PWRI, PWPP, and PR) in females, whereas no significant cross-lagged associations were observed in males. The prospective association between TG and ASI is particularly important. ASI has been shown to be a valuable indicator for assessing arterial stiffness (28). TG-induced endothelial injury and low-grade inflammation may impair arterial elasticity, directly promoting subsequent arterial stiffening. Therefore, our findings provide longitudinal evidence of a prospective association between TG and subsequent arterial stiffness. In addition, TG was prospectively associated with PWRI, PWPP, and PR. PWRI and PWPP are secondary DVP-derived parameters that reflect pulse wave characteristics, while PR is a pulse-related parameter. These findings suggest that the prospective association of TG may extend beyond arterial stiffness to other aspects of pulse-wave and pulse-related characteristics. However, further formal testing of between-sex differences indicated that only the prospective association between TG and PWRI differed significantly between sexes. The prospective association between TG and PWRI observed in females may be related to dynamic changes in estrogen levels. Hormonal changes associated with menopause can affect lipid metabolism, particularly by altering TG, LDL-C, and HDL-C levels (29); meanwhile, postmenopausal changes may impair arterial wave reflection and further accelerate arterial stiffening (30). Notably, previous evidence has indicated that this adverse vascular effect seems to persist among women receiving hormone replacement therapy (29). Future studies incorporating detailed information on menopausal status and hormone replacement therapy are warranted to further clarify their potential mechanism. We attribute these differences to several factors. First, our study population consisted of middle-aged and elderly adults, with an average age of 58 years or older. Previous studies have shown that approximately 40% of individuals over 65 years of age have dyslipidemia (31). Secondly, the measurement of arterial stiffness differed across studies: the previous two studies used brachial-ankle pulse wave velocity (baPWV) (26) and time-varying PWV (27), respectively, while our study employed DVP-derived ASI, PWRI, PWPP, and PR. These measures capture different aspects of arterial and pulse-wave characteristics and therefore may not be directly interchangeable.

In sensitivity analysis incorporating SBP, the previously directional cross-lagged associations were no longer statistically significant, suggesting that these associations may be sensitive to SBP. First, SBP may potentially mediate the association between lipid measures and arterial stiffness parameters. Previous research has demonstrated that SBP may mediate the association between remnant cholesterol and arterial stiffness in Chinese adults with hypertension (32). Therefore, incorporating SBP into our model may have attenuated the observed dyslipidemia-arterial stiffness prospective associations by accounting for part of this potential mediation pathway. Second, SBP may also act as a potential confounder. Previous studies have suggested a potential causal relationship between TG and both SBP and DBP (33), while higher blood pressure may contribute to the development and progression of arterial stiffness (34). Thus, incorporating SBP into the model may have reduced potential confounding, thereby attenuating the observed prospective associations. Third, SBP could theoretically act as a collider, and conditioning on it may introduce collider bias and alter the observed prospective associations. Future studies using longitudinal causal approaches are warranted to clarify the role of SBP in these relationships.

Mediation analysis showed no significant indirect effect through CRP, providing no evidence that CRP mediated the observed prospective associations between dyslipidemia and arterial stiffness. Inflammation has been proposed as a potential pathway linking dyslipidemia to arterial stiffness (35). These prospective associations may involve mechanisms beyond CRP-related inflammation, such as endothelial dysfunction, oxidative stress, and lipid accumulation, which are worthy of further investigation. Age-stratified analyses showed several significant prospective associations among participants aged <50 years but none among those aged ≥50 years. Formal interaction testing supported significant age differences for selected associations, including the associations of TG with ASI, LDL-C with ASI, and TC with PWPP. However, associations identified only in the age-stratified analyses without significant age interactions should be interpreted cautiously as exploratory findings.

Our study identified a prospective directional association between limited lipid measures and subsequent DVP-derived arterial stiffness parameters in predominantly middle-aged and elderly populations. Numerous studies have shown that various lipid indices are significantly associated with arterial stiffness (8, 10, 11). A cohort study demonstrated that individuals with elevated TyG indices and higher long-term trends are at greater risk for arterial stiffness (3). Another cohort study identified dyslipidemia as a determinant of arterial stiffness (17). Our findings indicate that the prospective associations were predominantly observed from lipid measures to subsequent vascular parameters, rather than vice versa. Furthermore, this pattern of association varies depending on the lipid measure, vascular parameter, and gender. These findings provide further longitudinal evidence of the relationship between dyslipidemia and subsequent arterial stiffness; however, further research is needed to elucidate the underlying mechanisms and clinical implications.

Increasing evidence shows the nonlinear association between clinical indicators and disease, for instance, the association between ASI and HDL-C with the risk of atherosclerotic cardiovascular disease is nonlinear (36, 37). The elaboration of nonlinear relationships helps to avoid the misconception that lower levels of clinical indicators such as TC, ApoB, and TG are better, and there is substantial support for nonlinear analysis (38). In our study, we further examined the prospective associations between dyslipidemia and arterial stiffness using the classical cross-lagged model and three nonlinear cross-lagged models (PRCM, GACM, and mixed-GACM), allowing both linear and nonlinear temporal patterns to be assessed.

Numerous studies have illustrated the impact of dyslipidemia on arterial stiffness, yet few have explored their prospective directional associations. In our study, we further examined these prospective associations using cross-lagged models. Our study also had several limitations. Firstly, the small sample size of participants in this study led to statistically unstable results. Secondly, according to the American Heart Association, arterial stiffness should be determined by measuring cfPWV (39). However, our study did not include cfPWV data. Thirdly, the cross-lagged analyses were restricted to participants who attended the first UKB repeat assessment, which may introduce selection bias. Although most baseline characteristics were broadly comparable between participants included in and excluded from the final analytical sample (Supplementary Table S1), caution is warranted when generalizing these quantitative estimates to broader populations. Fourthly, constrained by our two-wave study design, we were unable to fully distinguish stable between-person differences from within-person changes, requiring future studies with three or more measurement waves. Fifthly, the lack of physical activity data in our dataset precluded physical activity-stratified analyses and adjustment for its potential confounding effects, which may have resulted in residual confounding. Then, although SBP was incorporated as a third endogenous variable in a sensitivity analysis, residual time-varying confounding cannot be completely excluded. The previously associations were no longer statistically significant after accounting for SBP dynamics, suggesting that other time-varying factors may also influence the observed prospective directional associations. Finally, the participants in this study were middle-aged and elderly adults in the United Kingdom, so there may be limitations in extrapolating the results of this study to other ethnic groups and age groups.

## Conclusion

Our study found predominantly prospective directional associations from dyslipidemia to arterial stiffness, rather than vice versa. However, these associations were no longer statistically significant after incorporating SBP.

## Data Availability

The datasets used in this study were sourced from the UK Biobank. A comprehensive list of UK Biobank IDs for each index is provided in Supplementary material. Researchers can request access to the data via the UK Biobank’s official website (https://www.ukbiobank.ac.uk/).

## Glossary

CVD: Cardiovascular disease
HDL: High-density lipoprotein
LDL: Low-density lipoprotein
TC: Total cholesterol
TG: Triglycerides
HDL-C: High-density lipoprotein cholesterol
LDL-C: Low-density lipoprotein cholesterol
TyG: Triglyceride-glucose
ApoA: Apolipoprotein A
ApoB: Apolipoprotein B
LpA: Lipoprotein A
BMI: Body mass index
ALP: Alkaline phosphatase
ALT: Alanine aminotransferase
AST: Aspartate aminotransferase
GGT: Gamma-glutamyltransferase
TBIL: Total bilirubin
TP: Total protein
eGFR: Estimated glomerular filtration rate
CysC: Cystatin C
SHBG: Sex hormone binding globulin
CRP: C-reactive protein
VitD: Vitamin D
cfPWV: Carotid-femoral pulse wave velocity
PWRI: Pulse wave reflection index
PWPP: Pulse wave peak-to-peak
ASI: Arterial stiffness index
PR: Pulse rate
SBP: systolic blood pressure
RCS: Restricted cubic spline
PRCM: Polynomial regression-based cross-lagged model
GACM: Generalized additive cross-lagged model
mixed-GACM: Mixed generalized cross-lagged model
PWV: Pulse wave velocity
FDR: False discovery rate
DVP: digital volume pulse
EDF: effective degrees of freedom
