# Supplementary material for: Prospective directional associations between lipid measures and digital volume pulse-derived arterial stiffness parameters: evidence from cross-lagged panel analyses in the UK Biobank
Source: Front Nutr. 2026 Oct 29;13:1942971. doi: 10.3389/fnut.2026.1942971 (PMC13754874; doi:10.3389/fnut.2026.1942971)
Supplement: Supplementary file 1 [file Supplementary_file_1.docx]

**Supplementary Materials**

**Supplementary Tables:**

**Table S1. All indexes and corresponding UKB IDs.**

**Table S2. Descriptive Characteristics of Cohort Participants at Follow-up.**

**Table S3. Baseline Characteristics Between the Included and Excluded Participants**

**Table S4. Directional Cross-Lagged Associations Between Lipid Measures and Arterial Stiffness Parameters in Model 1**

**Table S5.** **Directional Cross-Lagged Associations Between Lipid Measures and Arterial Stiffness Parameters in Model 2.**

**Table S6. Formal Tests of Between-Sex Differences in the Main Directional Cross-Lagged Associations**

**Table S7. Mediation Analyses of Directional Cross-Lagged Associations Between Lipid Measures and Arterial Stiffness Parameters through C-Reactive Protein**

**Table S8. Age-Stratified Nonlinear Directional Cross-Lagged Associations Between Lipid Measures and Arterial Stiffness Parameters**

**Table S9. Formal Age-by-Variable Interaction Test for Directional Cross-Lagged Associations Between Lipid Measures and Arterial Stiffness Parameters**

**Table S10. Directional Cross-Lagged Associations Between Lipid Measures and Arterial Stiffness Parameters in the Mixed Generalized Additive Cross-lagged Model.**

**Table S11. Directional Cross-Lagged Associations Between Lipid Measures and Arterial Stiffness Parameters in the Polynomial Regression-based Cross-lagged Model.**

**Table S12. Sensitivity Analysis of GACM and Mixed-GACM Using Untransformed Variables**

**Table S13. Directional Cross-Lagged Associations Between Lipid Measures and Arterial Stiffness Parameters in the Generalized Additive Cross-lagged Model Incorporating SBP as a Third Endogenous Variable**

**Table S1. All indexes and corresponding** **UKB IDs.**

| **UKB ID**  **(****Field-Instance.Array)** | **Index** |
| --- | --- |
| 31-0.0 | Sex |
| 53-0.0; 53-1.0; 53-2.0; 53-3.0 | Date of attending assessment centre (Date) |
| 2443-0.0; 2443-1.0; 2443-2.0; 2443-3.0 | Diabetes diagnosed by doctor (Diabetes） |
| 4080-0.0, 4080-0.1; 4080-1.0, 4080-1.1; 4080-2.0, 4080-2.1; 4080-3.0, 4080-3.1 | Systolic blood pressure, automated reading (SBP) |
| 4194-0.0; 4194-1.0; 4194-2.0; 4194-3.0 | Pulse rate (PR) |
| 4195-0.0; 4195-1.0; 4195-2.0; 4195-3.0 | Pulse wave reflection index (PWRI) |
| 4196-0.0; 4196-1.0; 4196-2.0; 4196-3.0 | Pulse wave peak to peak time (PWPP) |
| 20116-0.0; 20116-1.0; 20116-2.0; 20116-3.0 | Smoking |
| 21001-0.0; 21001-1.0; 21001-2.0; 21001-3.0 | Body mass index (BMI) |
| 21003-0.0; 21003-1.0; 21003-2.0; 21003-3.0 | Age when attended assessment centre (Age) |
| 21021-0.0; 21021-1.0; 21021-2.0; 21021-3.0 | Pulse wave Arterial Stiffness index (ASI) |
| 23400-0.0; 23400-1.0 | Total Cholesterol (TC) |
| 23405-0.0; 23405-1.0 | LDL Cholesterol (LDL-C) |
| 23406-0.0; 23406-1.0 | HDL Cholesterol (HDL-C) |
| 30600-0.0; 30600-1.0 | Albumin |
| 30610-0.0; 30610-1.0 | Alkaline phosphatase (ALP) |
| 30620-0.0; 30620-1.0 | Alanine aminotransferase (ALT) |
| 30630-0.0; 30630-1.0 | Apolipoprotein A (ApoA) |
| 30640-0.0; 30640-1.0 | Apolipoprotein B (ApoB) |
| 30650-0.0; 30650-1.0 | Aspartate aminotransferase (AST) |
| 30670-0.0; 30670-1.0 | Urea |
| 30680-0.0; 30680-1.0 | Calcium |
| 30690-0.0; 30690-1.0 | Cholesterol |
| 30710-0.0; 30710-1.0 | C-reactive protein (CRP) |
| 30720-0.0; 30720-1.0 | Cystatin C (CysC) |
| 30730-0.0; 30730-1.0 | Gamma glutamyltransferase (GGT) |
| 30740-0.0; 30740-1.0 | Glucose |
| 30750-0.0; 30750-1.0 | Glycated haemoglobin (HbA1c) |
| 30770-0.0; 30770-1.0 | IGF-1 |
| 30790-0.0; 30790-1.0 | Lipoprotein A (LpA) |
| 30810-0.0; 30810-1.0 | Phosphate |
| 30830-0.0; 30830-1.0 | SHBG |
| 30840-0.0; 30840-1.0 | Total bilirubin (TBIL) |
| 30850-0.0; 30850-1.0 | Testosterone |
| 30860-0.0; 30860-1.0 | Total protein (TP) |
| 30870-0.0; 30870-1.0 | Triglycerides (TG) |
| 30880-0.0; 30880-1.0 | Urate |
| 30890-0.0; 30890-1.0 | Vitamin D (VitD) |
| 40005-(0-15).0 | Cancer |
| Note: UKB IDs are presented in the format “Field-Instance.Array”. For variables with multiple instances, the instance range is abbreviated (e.g., 40005-(0–15).0 means instances 0 to 15, all with array 0). | |

**Table S2. Descriptive Characteristics of Cohort Participants at Follow-up.**

| **Characteristics** | **Male (N=1,544)** | **Female (N=1,445)** | **P-value** |
| --- | --- | --- | --- |
| Age, years | 61.7 ± 7.2 | 60.4 ± 7.0 | <0.001 |
| Diabetes, n (%) | 113 (7.3) | 48 (3.3) | <0.001 |
| Smoking status, n (%) |  |  | <0.001 |
| Never | 834 (54.0) | 981 (67.9) |  |
| Previous | 634 (41.1) | 426 (29.5) |  |
| Current | 76 (4.9) | 38 (2.6) |  |
| SBP, mmHg | 140.5 ± 17.1 | 136.9 ± 19.4 | <0.001 |
| BMI | 27.2 ± 4.0 | 26.5 ± 4.9 | <0.001 |
| **Arterial Stiffness** |  |  |  |
| ASI, m/s | 10.1 (8.1 - 12.1) | 9.8 (7.8 - 11.5) | <0.001 |
| PWRI, % | 75.0 (68.0 - 81.0) | 72.0 (64.0 - 77.0) | <0.001 |
| PWPP, milliseconds | 173.5 (145.0 - 216.0) | 166.0 (141.0 - 206.0) | <0.001 |
| PR, bpm | 65.0 ± 11.3 | 66.8 ± 10.1 | <0.001 |
| log(PWPP) | 5.2 ± 0.3 | 5.1 ± 0.3 | <0.001 |
| log(PWRI) | 4.3 ± 0.2 | 4.2 ± 0.2 | <0.001 |
| **Glucose profiles** |  |  |  |
| Glucose, mmol/L | 5.0 (4.6 - 5.3) | 4.9 (4.6 - 5.2) | <0.001 |
| **Lipid profiles** |  |  |  |
| TC, mmol/L | 5.5 ± 1.2 | 6.0 ± 1.1 | <0.001 |
| HDL-C, mmol/L | 1.4 ± 0.3 | 1.7 ± 0.4 | <0.001 |
| LDL-C, mmol/L | 3.5 ± 0.9 | 3.7 ± 0.9 | <0.001 |
| TG, mmol/L | 1.6 (1.1 - 2.3) | 1.4 (1.0 - 1.9) | <0.001 |
| LpA, nmol/L | 29.7 (10.7 - 54.9) | 33.8 (12.7 - 55.9) | 0.062 |
| ApoA, g/L | 1.5 ± 0.2 | 1.7 ± 0.3 | <0.001 |
| ApoB, g/L | 1.0 ± 0.2 | 1.1 ± 0.2 | <0.001 |
| log(TG) | 0.5 ± 0.5 | 0.3 ± 0.5 | <0.001 |
| log(LpA) | 3.3± 1.0 | 3.4 ± 1.0 | 0.062 |
| **Liver function-related indexes** |  |  |  |
| Albumin, g/L | 45.8 ± 2.6 | 45.3 ± 2.6 | <0.001 |
| ALP, U/L | 79.1 (67.2 - 93.3) | 84.0 (70.3 - 99.5) | <0.001 |
| ALT, U/L | 22.7 (17.7 - 29.7) | 17.7 (14.5 - 23.0) | <0.001 |
| AST, U/L | 25.9 (22.5 - 30.3) | 23.7 (20.7 - 27.0) | <0.001 |
| GGT, U/L | 30.5 (22.1 - 44.0) | 21.2 (15.8 - 31.2) | <0.001 |
| Phosphate, mmol/L | 1.2 ± 0.1 | 1.3 ± 0.1 | <0.001 |
| TBIL, umol/L | 8.8 (7.1 - 11.2) | 7.1 (5.8 - 9.0) | <0.001 |
| TP, g/L | 73.3 ± 4.1 | 73.1 ± 4.1 | 0.115 |
| **Kidney function-related indexes** | |  |  |
| eGFR, mL/min/1.73 m^2^ | 88.6 ± 16.0 | 87.9 ± 15.6 | 0.230 |
| CysC, mg/L | 0.9 (0.8 - 1.0) | 0.9 (0.8 - 1.0) | <0.001 |
| Urea, U/L | 5.5 (4.8 - 6.3) | 5.3 (4.5 - 6.1) | <0.001 |
| Urate, umol/L | 350.2 (303.0 - 398.9) | 263.8 (226.5 - 307.0) | <0.001 |
| **Hormones** |  |  |  |
| IGF-1, nmol/L | 21.8 ± 5.2 | 20.6 ± 5.5 | <0.001 |
| Testosterone, nmol/L | 12.2 (10.0 - 14.7) | 1.1 (0.8 - 1.3) | <0.001 |
| SHBG, nmol/L | 42.3 (32.6 - 54.7) | 59.8 (43.7 - 80.6) | <0.001 |
| **Inflammatory factors** |  |  |  |
| CRP, mg/L | 1.2 (0.6 - 2.3) | 1.2 (0.6 - 2.5) | 0.297 |
| **Other biochemical indexes** |  |  |  |
| Calcium, mmol/L | 2.4 ± 0.1 | 2.4 ± 0.1 | <0.001 |
| VitD, nmol/L | 41.6 (29.5 - 56.2) | 44.5 (31.3 - 58.4) | 0.002 |
| Note: The values are mean (SD) and median (interquartile range) except for diabetes, smoking status in percentage.  Abbreviations: ASI, arterial stiffness index; PWRI, pulse wave reflection index; PWPP, pulse wave peak to peak time; PR, pulse rate; SBP, systolic blood pressure; TC, cholesterol; HDL-C, high-density lipoprotein cholesterol; LDL-C, low-density lipoprotein cholesterol; TG, triglycerides; LpA, lipoprotein A; ApoA, apolipoprotein A; ApoB, apolipoprotein B; BMI, Body mass index; ALP, alkaline phosphatase; ALT, alanine aminotransferase; AST, aspartate aminotransferase; GGT, gamma-glutamyltransferase; TBIL, total bilirubin; TP, total protein, eGFR, estimated glomerular filtration rate; CysC, cystatin C; SHBG, sex hormone binding globulin; CRP, c-reactive protein; VitD, vitamin D. | | | |

**Table S3. Baseline Characteristics Between the Included and Excluded** **Participants**

| **Characteristics** | **Included (N=2,989)** | **Excluded(N=17,355)** | **P-value** |
| --- | --- | --- | --- |
| Age, years | 58.1 ± 7.1 | 57.0 ± 7.5 | <0.001 |
| Male, n (%) | 1544 (51.7) | 8393 (48.4) | 0.001 |
| Diabetes, n (%) | 123 (4.1) | 703 (4.1) | 0.559 |
| Smoking status, n (%) |  |  | 0.031 |
| Never | 1791 (59.9) | 10178 (58.7) |  |
| Previous | 1037 (34.7) | 6006 (34.6) |  |
| Current | 153 (5.1) | 1130 (6.5) |  |
| SBP, mmHg | 141.7 ± 19.2 | 138.9 ± 19.2 | <0.001 |
| BMI | 26.9 ± 4.4 | 26.9 ± 4.5 | 0.722 |
| **Lipid profiles** |  |  |  |
| Cholesterol, mmol/L | 5.7 ± 1.1 | 5.7 ± 1.1 | 0.062 |
| HDL-C, mmol/L | 1.5 ± 0.4 | 1.5 ± 0.4 | 0.007 |
| LDL-C, mmol/L | 3.6 ± 0.9 | 3.5 ± 0.9 | 0.292 |
| TG, mmol/L | 1.5 (1.0 - 2.1) | 1.4 (1.0 - 2.1) | 0.036 |
| Note: The values are mean (SD) and median (interquartile range) except for diabetes, smoking status in percentage. | | | |
| Abbreviations: BMI, Body mass index; SBP, systolic blood pressure; HDL-C, high density lipoprotein cholesterol; LDL-C, low density lipoprotein cholesterol; TG, triglycerides; | | | |

**Table S4. Directional Cross-Lagged Associations Between Lipid Measures and Arterial Stiffness Parameters in Model 1**

| Association | Male | | | | | |  | Female | | | | | |
| --- | --- | --- | --- | --- | --- | --- | --- | --- | --- | --- | --- | --- | --- |
|  | *β* | 95% CI | SE | Z | Raw *P* | FDR *P* |  | *β* | 95% CI | SE | Z | Raw *P* | FDR *P* |
| ASI → ApoA | -0.037 | -0.071 – -0.004 | 0.017 | -2.211 | 0.027 | 0.054 |  | -0.011 | -0.047 – 0.025 | 0.018 | -0.613 | 0.540 | 0.917 |
| ApoA → ASI | -0.019 | -0.068 – 0.030 | 0.025 | -0.747 | 0.455 | 0.455 |  | -0.003 | -0.054 – 0.048 | 0.026 | -0.105 | 0.917 | 0.917 |
| ASI → ApoB | -0.003 | -0.039 – 0.033 | 0.018 | -0.156 | 0.876 | 0.876 |  | 0.028 | -0.005 – 0.061 | 0.017 | 1.637 | 0.102 | 0.149 |
| ApoB → ASI | 0.019 | -0.030 – 0.068 | 0.025 | 0.750 | 0.453 | 0.876 |  | 0.038 | -0.013 – 0.089 | 0.026 | 1.444 | 0.149 | 0.149 |
| ASI → HDL-C | -0.033 | -0.062 – -0.003 | 0.015 | -2.161 | 0.031 | 0.061 |  | -0.010 | -0.040 – 0.020 | 0.015 | -0.640 | 0.522 | 0.522 |
| HDL-C → ASI | -0.026 | -0.075 – 0.023 | 0.025 | -1.036 | 0.300 | 0.300 |  | -0.024 | -0.075 – 0.027 | 0.026 | -0.904 | 0.366 | 0.522 |
| ASI → LDL-C | 0.002 | -0.035 – 0.038 | 0.019 | 0.106 | 0.916 | 0.916 |  | 0.024 | -0.009 – 0.058 | 0.017 | 1.416 | 0.157 | 0.157 |
| LDL-C → ASI | 0.019 | -0.030 – 0.068 | 0.025 | 0.768 | 0.443 | 0.886 |  | 0.042 | -0.009 – 0.093 | 0.026 | 1.606 | 0.108 | 0.157 |
| ASI → log(LpA) | -0.012 | -0.041 – 0.018 | 0.015 | -0.766 | 0.444 | 0.753 |  | 0.016 | -0.017 – 0.048 | 0.017 | 0.936 | 0.349 | 0.503 |
| log(LpA) → ASI | -0.008 | -0.057 – 0.041 | 0.025 | -0.315 | 0.753 | 0.753 |  | -0.017 | -0.068 – 0.034 | 0.026 | -0.670 | 0.503 | 0.503 |
| ASI → log(TG) | 0.040 | 0.001 – 0.080 | 0.020 | 2.016 | 0.044 | 0.088 |  | 0.007 | -0.031 – 0.045 | 0.019 | 0.344 | 0.731 | 0.731 |
| log(TG) → ASI | 0.036 | -0.013 – 0.085 | 0.025 | 1.434 | 0.152 | 0.152 |  | 0.071 | 0.020 – 0.122 | 0.026 | 2.732 | 0.006 | 0.013 |
| ASI → TC | -0.007 | -0.043 – 0.029 | 0.018 | -0.378 | 0.706 | 0.706 |  | 0.019 | -0.015 – 0.053 | 0.018 | 1.077 | 0.282 | 0.282 |
| TC → ASI | 0.015 | -0.035 – 0.064 | 0.025 | 0.580 | 0.562 | 0.706 |  | 0.037 | -0.014 – 0.088 | 0.026 | 1.413 | 0.158 | 0.282 |
| log(PWRI) → ApoA | -0.031 | -0.065 – 0.002 | 0.017 | -1.859 | 0.063 | 0.126 |  | 0.022 | -0.013 – 0.058 | 0.018 | 1.225 | 0.220 | 0.441 |
| ApoA → log(PWRI) | 0.032 | -0.017 – 0.082 | 0.025 | 1.278 | 0.201 | 0.201 |  | 0.006 | -0.045 – 0.058 | 0.026 | 0.247 | 0.805 | 0.805 |
| log(PWRI) → ApoB | 0.000 | -0.036 – 0.036 | 0.018 | 0.009 | 0.993 | 0.993 |  | 0.005 | -0.028 – 0.038 | 0.017 | 0.308 | 0.758 | 0.758 |
| ApoB → log(PWRI) | -0.057 | -0.106 – -0.007 | 0.025 | -2.248 | 0.025 | 0.049 |  | 0.028 | -0.023 – 0.079 | 0.026 | 1.062 | 0.288 | 0.577 |
| log(PWRI) → HDL-C | -0.028 | -0.057 – 0.002 | 0.015 | -1.819 | 0.069 | 0.138 |  | 0.009 | -0.021 – 0.038 | 0.015 | 0.559 | 0.576 | 0.647 |
| HDL-C → log(PWRI) | 0.036 | -0.014 – 0.085 | 0.025 | 1.412 | 0.158 | 0.158 |  | 0.012 | -0.039 – 0.063 | 0.026 | 0.458 | 0.647 | 0.647 |
| log(PWRI) → LDL-C | 0.000 | -0.037 – 0.036 | 0.019 | -0.012 | 0.990 | 0.990 |  | -0.002 | -0.035 – 0.032 | 0.017 | -0.096 | 0.923 | 0.923 |
| LDL-C → log(PWRI) | -0.039 | -0.088 – 0.011 | 0.025 | -1.522 | 0.128 | 0.256 |  | 0.038 | -0.013 – 0.089 | 0.026 | 1.450 | 0.147 | 0.294 |
| log(PWRI) → log(LpA) | -0.008 | -0.038 – 0.021 | 0.015 | -0.546 | 0.585 | 0.585 |  | 0.009 | -0.023 – 0.042 | 0.017 | 0.558 | 0.577 | 0.577 |
| log(LpA) → log(PWRI) | 0.039 | -0.010 – 0.089 | 0.025 | 1.554 | 0.120 | 0.241 |  | -0.040 | -0.091 – 0.011 | 0.026 | -1.521 | 0.128 | 0.256 |
| log(PWRI) → log(TG) | 0.019 | -0.020 – 0.058 | 0.020 | 0.947 | 0.344 | 0.344 |  | -0.013 | -0.051 – 0.025 | 0.019 | -0.653 | 0.514 | 0.514 |
| log(TG) → log(PWRI) | -0.051 | -0.101 – -0.002 | 0.025 | -2.021 | 0.043 | 0.086 |  | 0.052 | 0.001 – 0.103 | 0.026 | 2.013 | 0.044 | 0.088 |
| log(PWRI) → TC | -0.004 | -0.041 – 0.032 | 0.018 | -0.243 | 0.808 | 0.808 |  | 0.000 | -0.035 – 0.034 | 0.018 | -0.010 | 0.992 | 0.992 |
| TC → log(PWRI) | -0.031 | -0.080 – 0.019 | 0.025 | -1.210 | 0.226 | 0.453 |  | 0.042 | -0.009 – 0.093 | 0.026 | 1.625 | 0.104 | 0.208 |
| log(PWPP) → ApoA | 0.033 | -0.001 – 0.066 | 0.017 | 1.921 | 0.055 | 0.109 |  | 0.009 | -0.027 – 0.045 | 0.018 | 0.487 | 0.626 | 0.789 |
| ApoA → log(PWPP) | 0.010 | -0.039 – 0.059 | 0.025 | 0.389 | 0.698 | 0.698 |  | -0.007 | -0.058 – 0.044 | 0.026 | -0.267 | 0.789 | 0.789 |
| log(PWPP) → ApoB | 0.008 | -0.028 – 0.044 | 0.018 | 0.415 | 0.678 | 0.678 |  | -0.034 | -0.067 – -0.000 | 0.017 | -1.980 | 0.048 | 0.095 |
| ApoB → log(PWPP) | -0.030 | -0.079 – 0.019 | 0.025 | -1.188 | 0.235 | 0.470 |  | -0.044 | -0.095 – 0.008 | 0.026 | -1.669 | 0.095 | 0.095 |
| log(PWPP) → HDL-C | 0.024 | -0.006 – 0.054 | 0.015 | 1.590 | 0.112 | 0.224 |  | 0.010 | -0.020 – 0.040 | 0.015 | 0.629 | 0.530 | 0.625 |
| HDL-C → log(PWPP) | 0.012 | -0.037 – 0.061 | 0.025 | 0.496 | 0.620 | 0.620 |  | 0.013 | -0.038 – 0.064 | 0.026 | 0.489 | 0.625 | 0.625 |
| log(PWPP) → LDL-C | 0.004 | -0.032 – 0.041 | 0.019 | 0.218 | 0.828 | 0.828 |  | -0.031 | -0.065 – 0.002 | 0.017 | -1.821 | 0.069 | 0.074 |
| LDL-C → log(PWPP) | -0.027 | -0.076 – 0.022 | 0.025 | -1.094 | 0.274 | 0.548 |  | -0.047 | -0.098 – 0.005 | 0.026 | -1.784 | 0.074 | 0.074 |
| log(PWPP) → log(LpA) | 0.013 | -0.016 – 0.042 | 0.015 | 0.863 | 0.388 | 0.617 |  | -0.019 | -0.051 – 0.014 | 0.017 | -1.123 | 0.261 | 0.368 |
| log(LpA) → log(PWPP) | 0.013 | -0.036 – 0.061 | 0.025 | 0.501 | 0.617 | 0.617 |  | 0.023 | -0.028 – 0.075 | 0.026 | 0.900 | 0.368 | 0.368 |
| log(PWPP) → log(TG) | -0.033 | -0.072 – 0.007 | 0.020 | -1.632 | 0.103 | 0.205 |  | -0.004 | -0.042 – 0.034 | 0.019 | -0.211 | 0.833 | 0.833 |
| log(TG) → log(PWPP) | -0.030 | -0.079 – 0.019 | 0.025 | -1.183 | 0.237 | 0.237 |  | -0.066 | -0.117 – -0.015 | 0.026 | -2.532 | 0.011 | 0.023 |
| log(PWPP) → TC | 0.011 | -0.026 – 0.047 | 0.018 | 0.581 | 0.561 | 0.561 |  | -0.025 | -0.059 – 0.010 | 0.018 | -1.418 | 0.156 | 0.156 |
| TC → log(PWPP) | -0.023 | -0.072 – 0.026 | 0.025 | -0.926 | 0.355 | 0.561 |  | -0.044 | -0.095 – 0.007 | 0.026 | -1.698 | 0.090 | 0.156 |
| PR → ApoA | 0.012 | -0.021 – 0.045 | 0.017 | 0.703 | 0.482 | 0.482 |  | -0.011 | -0.046 – 0.025 | 0.018 | -0.578 | 0.563 | 0.980 |
| ApoA → PR | -0.020 | -0.059 – 0.020 | 0.020 | -0.973 | 0.331 | 0.482 |  | 0.001 | -0.040 – 0.041 | 0.021 | 0.025 | 0.980 | 0.980 |
| PR → ApoB | 0.026 | -0.010 – 0.062 | 0.018 | 1.415 | 0.157 | 0.157 |  | 0.017 | -0.016 – 0.051 | 0.017 | 1.032 | 0.302 | 0.604 |
| ApoB → PR | 0.035 | -0.004 – 0.074 | 0.020 | 1.751 | 0.080 | 0.157 |  | -0.003 | -0.044 – 0.038 | 0.021 | -0.161 | 0.872 | 0.872 |
| PR → HDL-C | -0.004 | -0.034 – 0.026 | 0.015 | -0.267 | 0.789 | 0.789 |  | -0.007 | -0.037 – 0.023 | 0.015 | -0.458 | 0.647 | 0.647 |
| HDL-C → PR | -0.020 | -0.060 – 0.019 | 0.020 | -1.013 | 0.311 | 0.622 |  | -0.018 | -0.059 – 0.023 | 0.021 | -0.849 | 0.396 | 0.647 |
| PR → LDL-C | 0.026 | -0.011 – 0.062 | 0.019 | 1.381 | 0.167 | 0.329 |  | 0.016 | -0.017 – 0.050 | 0.017 | 0.958 | 0.338 | 0.676 |
| LDL-C → PR | 0.020 | -0.020 – 0.059 | 0.020 | 0.977 | 0.329 | 0.329 |  | -0.008 | -0.049 – 0.033 | 0.021 | -0.372 | 0.710 | 0.710 |
| PR → log(LpA) | -0.004 | -0.034 – 0.025 | 0.015 | -0.282 | 0.778 | 0.778 |  | 0.007 | -0.025 – 0.040 | 0.017 | 0.440 | 0.660 | 0.660 |
| log(LpA) → PR | -0.020 | -0.059 – 0.020 | 0.020 | -0.984 | 0.325 | 0.650 |  | -0.014 | -0.055 – 0.027 | 0.021 | -0.690 | 0.490 | 0.660 |
| PR → log(TG) | -0.002 | -0.042 – 0.038 | 0.020 | -0.117 | 0.907 | 0.907 |  | 0.002 | -0.036 – 0.041 | 0.020 | 0.123 | 0.902 | 0.902 |
| log(TG) → PR | 0.007 | -0.033 – 0.047 | 0.020 | 0.348 | 0.728 | 0.907 |  | -0.027 | -0.068 – 0.014 | 0.021 | -1.295 | 0.195 | 0.391 |
| PR → TC | 0.014 | -0.022 – 0.050 | 0.018 | 0.750 | 0.453 | 0.466 |  | 0.008 | -0.026 – 0.043 | 0.018 | 0.472 | 0.637 | 0.637 |
| TC → PR | 0.015 | -0.025 – 0.054 | 0.020 | 0.730 | 0.466 | 0.466 |  | -0.016 | -0.056 – 0.025 | 0.021 | -0.742 | 0.458 | 0.637 |
| Note: *β* indicates the effect size for the cross-lagged regression. SE indicates the standard error of the cross-lagged regression. Benjamini-Hochberg false discovery rate-corrected (FDR) *P*-value for multiple testing. Analyses were adjusted for Age, Smoking, Diabetes, SBP, Glucose, eGFR, CRP, IGF1, BMI, Albumin, ALP, ALT, AST, GGT, Phosphate, TBIL, TP, CysC, Urea, Urate, Testosterone, SHBG, Calcium and VitD at baseline. For categorical variables: the p-value was calculated using the Fisher exact test; for normally distributed continuous variables: the p-value was calculated using a t-test; for non-normally distributed continuous variables, the p-value was calculated using the Wilcoxon rank sum test. | | | | | | | | | | | | | |
| Abbreviations: ASI, arterial stiffness index; PWRI, pulse wave reflection index; PWPP, pulse wave peak to peak time; PR, pulse rate; SBP, systolic blood pressure; TC, cholesterol; HDL-C, high-density lipoprotein cholesterol; LDL-C, low-density lipoprotein cholesterol; TG, triglycerides; LpA, lipoprotein A; ApoA, apolipoprotein A; ApoB, apolipoprotein B; BMI, Body mass index; ALP, alkaline phosphatase; ALT, alanine aminotransferase; AST, aspartate aminotransferase; GGT, gamma-glutamyltransferase; TBIL, total bilirubin; TP, total protein, eGFR, estimated glomerular filtration rate; CysC, cystatin C; SHBG, sex hormone binding globulin; CRP, c-reactive protein; VitD, vitamin D. | | | | | | | | | | | | | |

**Table S5. Directional Cross-Lagged Associations Between Lipid Measures and Arterial Stiffness Parameters in Model 2**

| Association | Male | | | | | |  | Female | | | | | |
| --- | --- | --- | --- | --- | --- | --- | --- | --- | --- | --- | --- | --- | --- |
|  | *β* | 95% CI | SE | Z | Raw *P* | FDR *P* |  | *β* | 95% CI | SE | Z | Raw *P* | FDR *P* |
| ASI → ApoA | -0.031 | -0.063 – 0.002 | 0.017 | -1.851 | 0.064 | 0.128 |  | -0.019 | -0.055 – 0.017 | 0.018 | -1.044 | 0.296 | 0.593 |
| ApoA → ASI | -0.006 | -0.055 – 0.043 | 0.025 | -0.230 | 0.818 | 0.818 |  | 0.000 | -0.051 – 0.051 | 0.026 | -0.010 | 0.992 | 0.992 |
| ASI → ApoB | 0.002 | -0.034 – 0.037 | 0.018 | 0.088 | 0.930 | 0.930 |  | 0.021 | -0.012 – 0.053 | 0.017 | 1.238 | 0.216 | 0.250 |
| ApoB → ASI | 0.014 | -0.035 – 0.064 | 0.025 | 0.573 | 0.567 | 0.930 |  | 0.030 | -0.021 – 0.081 | 0.026 | 1.151 | 0.250 | 0.250 |
| ASI → HDL-C | -0.024 | -0.053 – 0.006 | 0.015 | -1.593 | 0.111 | 0.222 |  | -0.015 | -0.046 – 0.016 | 0.016 | -0.959 | 0.338 | 0.523 |
| HDL-C → ASI | -0.012 | -0.061 – 0.037 | 0.025 | -0.486 | 0.627 | 0.627 |  | -0.017 | -0.068 – 0.034 | 0.026 | -0.638 | 0.523 | 0.523 |
| ASI → LDL-C | 0.008 | -0.028 – 0.043 | 0.018 | 0.429 | 0.668 | 0.668 |  | 0.019 | -0.014 – 0.052 | 0.017 | 1.111 | 0.266 | 0.266 |
| LDL-C → ASI | 0.017 | -0.032 – 0.066 | 0.025 | 0.690 | 0.490 | 0.668 |  | 0.034 | -0.017 – 0.085 | 0.026 | 1.309 | 0.191 | 0.266 |
| ASI → log(LpA) | -0.008 | -0.038 – 0.022 | 0.015 | -0.525 | 0.599 | 0.685 |  | 0.017 | -0.016 – 0.050 | 0.017 | 1.006 | 0.314 | 0.482 |
| log(LpA) → ASI | -0.010 | -0.059 – 0.039 | 0.025 | -0.405 | 0.685 | 0.685 |  | -0.018 | -0.069 – 0.033 | 0.026 | -0.703 | 0.482 | 0.482 |
| ASI → log(TG) | 0.027 | -0.014 – 0.068 | 0.021 | 1.311 | 0.190 | 0.379 |  | 0.004 | -0.037 – 0.044 | 0.021 | 0.175 | 0.861 | 0.861 |
| log(TG) → ASI | 0.020 | -0.030 – 0.069 | 0.025 | 0.779 | 0.436 | 0.436 |  | 0.052 | 0.002 – 0.103 | 0.026 | 2.022 | 0.043 | 0.086 |
| ASI → TC | -0.002 | -0.037 – 0.034 | 0.018 | -0.098 | 0.922 | 0.922 |  | 0.014 | -0.020 – 0.047 | 0.017 | 0.799 | 0.424 | 0.424 |
| TC → ASI | 0.013 | -0.036 – 0.063 | 0.025 | 0.535 | 0.593 | 0.922 |  | 0.030 | -0.021 – 0.081 | 0.026 | 1.160 | 0.246 | 0.424 |
| log(PWRI) → ApoA | -0.025 | -0.058 – 0.007 | 0.017 | -1.522 | 0.128 | 0.219 |  | 0.006 | -0.030 – 0.042 | 0.018 | 0.317 | 0.752 | 0.885 |
| ApoA → log(PWRI) | 0.031 | -0.019 – 0.081 | 0.025 | 1.228 | 0.219 | 0.219 |  | 0.004 | -0.047 – 0.055 | 0.026 | 0.144 | 0.885 | 0.885 |
| log(PWRI) → ApoB | 0.002 | -0.033 – 0.037 | 0.018 | 0.090 | 0.928 | 0.928 |  | 0.008 | -0.024 – 0.041 | 0.017 | 0.501 | 0.616 | 0.616 |
| ApoB → log(PWRI) | -0.054 | -0.103 – -0.004 | 0.025 | -2.116 | 0.034 | 0.069 |  | 0.042 | -0.010 – 0.093 | 0.026 | 1.594 | 0.111 | 0.222 |
| log(PWRI) → HDL-C | -0.022 | -0.052 – 0.007 | 0.015 | -1.475 | 0.140 | 0.208 |  | -0.008 | -0.038 – 0.023 | 0.016 | -0.497 | 0.619 | 0.847 |
| HDL-C → log(PWRI) | 0.032 | -0.018 – 0.082 | 0.025 | 1.260 | 0.208 | 0.208 |  | 0.005 | -0.046 – 0.056 | 0.026 | 0.193 | 0.847 | 0.847 |
| log(PWRI) → LDL-C | 0.001 | -0.035 – 0.036 | 0.018 | 0.038 | 0.969 | 0.969 |  | 0.001 | -0.032 – 0.034 | 0.017 | 0.035 | 0.972 | 0.972 |
| LDL-C → log(PWRI) | -0.037 | -0.087 – 0.013 | 0.025 | -1.463 | 0.143 | 0.287 |  | 0.051 | -0.000 – 0.102 | 0.026 | 1.947 | 0.052 | 0.103 |
| log(PWRI) → log(LpA) | -0.009 | -0.038 – 0.021 | 0.015 | -0.562 | 0.574 | 0.574 |  | 0.008 | -0.025 – 0.041 | 0.017 | 0.495 | 0.621 | 0.621 |
| log(LpA) → log(PWRI) | 0.034 | -0.016 – 0.083 | 0.025 | 1.330 | 0.183 | 0.367 |  | -0.042 | -0.093 – 0.009 | 0.026 | -1.611 | 0.107 | 0.214 |
| log(PWRI) → log(TG) | 0.014 | -0.027 – 0.054 | 0.021 | 0.651 | 0.515 | 0.515 |  | -0.001 | -0.041 – 0.040 | 0.021 | -0.040 | 0.968 | 0.968 |
| log(TG) → log(PWRI) | -0.041 | -0.091 – 0.009 | 0.025 | -1.621 | 0.105 | 0.210 |  | 0.071 | 0.020 – 0.122 | 0.026 | 2.745 | 0.006 | 0.012 |
| log(PWRI) → TC | -0.003 | -0.039 – 0.032 | 0.018 | -0.190 | 0.849 | 0.849 |  | -0.004 | -0.037 – 0.030 | 0.017 | -0.227 | 0.821 | 0.821 |
| TC → log(PWRI) | -0.030 | -0.080 – 0.019 | 0.025 | -1.194 | 0.232 | 0.465 |  | 0.054 | 0.003 – 0.105 | 0.026 | 2.073 | 0.038 | 0.076 |
| log(PWPP) → ApoA | 0.028 | -0.005 – 0.060 | 0.017 | 1.675 | 0.094 | 0.188 |  | 0.022 | -0.014 – 0.058 | 0.018 | 1.218 | 0.223 | 0.447 |
| ApoA → log(PWPP) | -0.003 | -0.052 – 0.046 | 0.025 | -0.113 | 0.910 | 0.910 |  | -0.010 | -0.061 – 0.042 | 0.026 | -0.367 | 0.714 | 0.714 |
| log(PWPP) → ApoB | 0.005 | -0.030 – 0.040 | 0.018 | 0.282 | 0.778 | 0.778 |  | -0.025 | -0.057 – 0.008 | 0.017 | -1.486 | 0.137 | 0.175 |
| ApoB → log(PWPP) | -0.025 | -0.074 – 0.025 | 0.025 | -0.980 | 0.327 | 0.654 |  | -0.035 | -0.087 – 0.016 | 0.026 | -1.356 | 0.175 | 0.175 |
| log(PWPP) → HDL-C | 0.015 | -0.014 – 0.044 | 0.015 | 1.005 | 0.315 | 0.630 |  | 0.019 | -0.011 – 0.050 | 0.016 | 1.225 | 0.221 | 0.441 |
| HDL-C → log(PWPP) | -0.001 | -0.050 – 0.048 | 0.025 | -0.039 | 0.969 | 0.969 |  | 0.007 | -0.044 – 0.058 | 0.026 | 0.261 | 0.794 | 0.794 |
| log(PWPP) → LDL-C | 0.000 | -0.036 – 0.035 | 0.018 | -0.003 | 0.998 | 0.998 |  | -0.023 | -0.056 – 0.010 | 0.017 | -1.387 | 0.165 | 0.165 |
| LDL-C → log(PWPP) | -0.024 | -0.074 – 0.025 | 0.025 | -0.977 | 0.329 | 0.657 |  | -0.037 | -0.089 – 0.014 | 0.026 | -1.434 | 0.151 | 0.165 |
| log(PWPP) → log(LpA) | 0.010 | -0.020 – 0.040 | 0.015 | 0.671 | 0.502 | 0.535 |  | -0.019 | -0.052 – 0.014 | 0.017 | -1.148 | 0.251 | 0.363 |
| log(LpA) → log(PWPP) | 0.016 | -0.034 – 0.065 | 0.025 | 0.621 | 0.535 | 0.535 |  | 0.024 | -0.027 – 0.075 | 0.026 | 0.909 | 0.363 | 0.363 |
| log(PWPP) → log(TG) | -0.019 | -0.060 – 0.022 | 0.021 | -0.917 | 0.359 | 0.570 |  | -0.002 | -0.042 – 0.039 | 0.021 | -0.085 | 0.932 | 0.932 |
| log(TG) → log(PWPP) | -0.014 | -0.063 – 0.035 | 0.025 | -0.568 | 0.570 | 0.570 |  | -0.050 | -0.101 – 0.001 | 0.026 | -1.915 | 0.056 | 0.111 |
| log(PWPP) → TC | 0.007 | -0.028 – 0.042 | 0.018 | 0.392 | 0.695 | 0.695 |  | -0.016 | -0.050 – 0.017 | 0.017 | -0.948 | 0.343 | 0.343 |
| TC → log(PWPP) | -0.021 | -0.070 – 0.028 | 0.025 | -0.852 | 0.394 | 0.695 |  | -0.036 | -0.087 – 0.015 | 0.026 | -1.389 | 0.165 | 0.330 |
| PR → ApoA | 0.017 | -0.016 – 0.049 | 0.017 | 1.006 | 0.314 | 0.441 |  | 0.002 | -0.034 – 0.037 | 0.018 | 0.083 | 0.934 | 0.934 |
| ApoA → PR | -0.016 | -0.055 – 0.024 | 0.020 | -0.770 | 0.441 | 0.441 |  | 0.017 | -0.025 – 0.058 | 0.021 | 0.782 | 0.434 | 0.869 |
| PR → ApoB | 0.010 | -0.025 – 0.045 | 0.018 | 0.564 | 0.573 | 0.573 |  | 0.010 | -0.022 – 0.043 | 0.017 | 0.608 | 0.543 | 0.543 |
| ApoB → PR | 0.021 | -0.019 – 0.061 | 0.020 | 1.044 | 0.296 | 0.573 |  | -0.016 | -0.058 – 0.026 | 0.021 | -0.755 | 0.450 | 0.543 |
| PR → HDL-C | 0.007 | -0.023 – 0.036 | 0.015 | 0.442 | 0.658 | 0.658 |  | 0.009 | -0.022 – 0.039 | 0.016 | 0.553 | 0.580 | 0.803 |
| HDL-C → PR | -0.011 | -0.050 – 0.029 | 0.020 | -0.528 | 0.598 | 0.658 |  | 0.005 | -0.036 – 0.047 | 0.021 | 0.250 | 0.803 | 0.803 |
| PR → LDL-C | 0.011 | -0.024 – 0.047 | 0.018 | 0.617 | 0.537 | 0.574 |  | 0.011 | -0.022 – 0.044 | 0.017 | 0.669 | 0.503 | 0.503 |
| LDL-C → PR | 0.011 | -0.028 – 0.051 | 0.020 | 0.562 | 0.574 | 0.574 |  | -0.019 | -0.061 – 0.022 | 0.021 | -0.911 | 0.362 | 0.503 |
| PR → log(LpA) | 0.001 | -0.029 – 0.030 | 0.015 | 0.034 | 0.973 | 0.973 |  | 0.010 | -0.022 – 0.043 | 0.017 | 0.616 | 0.538 | 0.689 |
| log(LpA) → PR | -0.015 | -0.055 – 0.025 | 0.020 | -0.724 | 0.469 | 0.938 |  | -0.009 | -0.050 – 0.033 | 0.021 | -0.400 | 0.689 | 0.689 |
| PR → log(TG) | -0.012 | -0.053 – 0.029 | 0.021 | -0.569 | 0.569 | 0.917 |  | -0.015 | -0.056 – 0.026 | 0.021 | -0.728 | 0.466 | 0.466 |
| log(TG) → PR | 0.002 | -0.038 – 0.042 | 0.020 | 0.104 | 0.917 | 0.917 |  | -0.053 | -0.094 – -0.011 | 0.021 | -2.469 | 0.014 | 0.027 |
| PR → TC | 0.006 | -0.030 – 0.041 | 0.018 | 0.318 | 0.750 | 0.750 |  | 0.010 | -0.024 – 0.043 | 0.017 | 0.584 | 0.559 | 0.559 |
| TC → PR | 0.010 | -0.030 – 0.049 | 0.020 | 0.477 | 0.634 | 0.750 |  | -0.022 | -0.063 – 0.020 | 0.021 | -1.021 | 0.307 | 0.559 |
| Note: *β* indicates the effect size for the cross-lagged regression. SE indicates the standard error of the cross-lagged regression. Benjamini-Hochberg false discovery rate-corrected (FDR) P-values for multiple testing. *β* and their corresponding 95% confidence intervals (CIs) were reported alongside raw P-values and FDR P-values. Analyses were adjusted for Age, Smoking, Diabetes, SBP, Glucose, eGFR, CRP, IGF1, BMI, Albumin, ALP, ALT, AST, GGT, Phosphate, TBIL, TP, CysC, Urea, Urate, Testosterone, SHBG, Calcium and VitD at baseline. For categorical variables: the p-value was calculated using the Fisher exact test; for normally distributed continuous variables: the p-value was calculated using a t-test; for non-normally distributed continuous variables, the p-value was calculated using the Wilcoxon rank sum test. | | | | | | | | | | | | | |
| Abbreviations: ASI, arterial stiffness index; PWRI, pulse wave reflection index; PWPP, pulse wave peak to peak time; PR, pulse rate; SBP, systolic blood pressure; TC, cholesterol; HDL-C, high-density lipoprotein cholesterol; LDL-C, low-density lipoprotein cholesterol; TG, triglycerides; LpA, lipoprotein A; ApoA, apolipoprotein A; ApoB, apolipoprotein B; BMI, Body mass index; ALP, alkaline phosphatase; ALT, alanine aminotransferase; AST, aspartate aminotransferase; GGT, gamma-glutamyltransferase; TBIL, total bilirubin; TP, total protein, eGFR, estimated glomerular filtration rate; CysC, cystatin C; SHBG, sex hormone binding globulin; CRP, c-reactive protein; VitD, vitamin D. | | | | | | | | | | | | | |

**Table S6. Formal Tests of Between-Sex Differences in the Main Directional Cross-Lagged Associations**

| Association | β (Male) | β (Female) | Sex difference *P* |
| --- | --- | --- | --- |
| ApoB → PWRI | -0.057 | 0.028 | 0.020 |
| TG → ASI | 0.036 | 0.071 | 0.332 |
| TG → PWRI | -0.051 | 0.052 | 0.004 |
| TG → PWPP | -0.030 | -0.066 | 0.314 |
| TG → PR | 0.007 | -0.027 | 0.242 |
| Note: *β* indicates the standardized cross-lagged regression coefficient. *P* for sex difference was obtained from Wald tests comparing the corresponding cross-lagged paths between males and females using multi-group structural equation modeling.  Abbreviations: ASI, arterial stiffness index; ApoB, apolipoprotein B; PWRI, pulse wave reflection index; PWPP, pulse wave peak to peak time; PR, pulse rate; TG, triglycerides. | | | |

**Table S7. Mediation Analyses of Directional Cross-Lagged Associations Between Lipid Measures and Arterial Stiffness Parameters through C-Reactive Protein**

| **Pathway** | **Effect** | **β** | **95% CI** | ***P* value** |
| --- | --- | --- | --- | --- |
| **Male** |  |  |  |  |
| ApoB → CRP → PWRI | Indirect | 0.158 | -0.188 – 0.842 | 0.497 |
|  | Direct | -8.841 | -23.164 – 0.760 | 0.094 |
|  | Total | -8.683 | -22.578 – 0.731 | 0.088 |
| **Female** |  |  |  |  |
| TG → CRP → ASI | Indirect | 0.006 | -0.002 – 0.020 | 0.220 |
|  | Direct | 0.308 | 0.085 – 0.567 | 0.004 |
|  | Total | 0.314 | 0.089 – 0.573 | 0.004 |
| TG → CRP → PWRI | Indirect | 0.006 | -0.031 – 0.052 | 0.766 |
|  | Direct | 1.700 | -0.067 – 4.592 | 0.064 |
|  | Total | 1.706 | -0.071 – 4.619 | 0.066 |
| TG → CRP → PWPP | Indirect | -0.095 | -0.339 – 0.044 | 0.244 |
|  | Direct | -4.047 | -9.081 – 0.853 | 0.110 |
|  | Total | -4.143 | -9.162 – 0.756 | 0.099 |
| TG → CRP → PR | Indirect | 0.037 | -0.013 – 0.096 | 0.162 |
|  | Direct | -0.426 | -1.003 – 0.210 | 0.173 |
|  | Total | -0.389 | -0.968 – 0.254 | 0.216 |
| Note: Effect estimates are presented as β with corresponding 95% CIs. Indirect, direct, and total effects were estimated using 5,000 bootstrap resamples. P-values were calculated as empirical two-sided P-values based on the bootstrap distributions.  Abbreviations: ASI, arterial stiffness index; ApoB, apolipoprotein B; CRP indicates C-reactive protein; PWRI, pulse wave reflection index; PWPP, pulse wave peak to peak time; PR, pulse rate; TG, triglycerides. | | | | |

**Table S8. Age-Stratified Nonlinear Directional Cross-Lagged Associations Between Lipid Measures and Arterial Stiffness Parameters**

| Association | Age < 50 | | | |  | Age >=50 | | | |
| --- | --- | --- | --- | --- | --- | --- | --- | --- | --- |
|  | EDF | F | Raw *P* | FDR *P* |  | EDF | F | Raw *P* | FDR *P* |
| ApoA   → ASI | 3.623 | 1.066 | 0.337 | 0.337 |  | 5.156 | 0.984 | 0.426 | 0.426 |
| ApoB   → ASI | 4.849 | 2.248 | 0.038 | 0.076 |  | 1.000 | 0.351 | 0.554 | 0.576 |
| HDL_C   → ASI | 1.000 | 0.916 | 0.339 | 0.339 |  | 2.650 | 0.417 | 0.689 | 0.689 |
| LDL_C   → ASI | 1.484 | 3.767 | 0.019 | 0.037 |  | 1.000 | 0.454 | 0.500 | 0.500 |
| LpA   → ASI | 1.385 | 0.588 | 0.646 | 0.646 |  | 1.000 | 0.354 | 0.552 | 0.552 |
| TG   → ASI | 6.043 | 3.604 | <0.001 | 0.002 |  | 2.554 | 1.475 | 0.203 | 0.406 |
| TC   → ASI | 1.058 | 4.169 | 0.043 | 0.086 |  | 1.243 | 0.266 | 0.622 | 0.738 |
| ApoA   → PWRI | 1.000 | 1.833 | 0.176 | 0.353 |  | 1.000 | 0.077 | 0.781 | 0.781 |
| ApoB   → PWRI | 7.879 | 6.732 | <0.001 | <0.001 |  | 1.000 | 1.335 | 0.248 | 0.496 |
| HDL C   → PWRI | 1.000 | 0.803 | 0.371 | 0.480 |  | 1.000 | 0.002 | 0.964 | 0.964 |
| LDL C   → PWRI | 2.052 | 2.356 | 0.105 | 0.210 |  | 1.000 | 0.759 | 0.384 | 0.515 |
| LpA   → PWRI | 1.000 | 0.899 | 0.344 | 0.344 |  | 1.199 | 0.073 | 0.818 | 0.818 |
| TG   → PWRI | 1.000 | 0.004 | 0.947 | 0.947 |  | 5.117 | 0.758 | 0.657 | 0.657 |
| TC   → PWRI | 2.346 | 2.099 | 0.093 | 0.186 |  | 1.000 | 0.434 | 0.510 | 0.613 |
| ApoA   → PWPP | 3.401 | 0.776 | 0.540 | 0.540 |  | 1.000 | 0.114 | 0.736 | 0.736 |
| ApoB   → PWPP | 7.434 | 5.097 | <0.001 | <0.001 |  | 1.000 | 1.330 | 0.249 | 0.498 |
| HDL C   → PWPP | 1.000 | 0.000 | 0.986 | 0.986 |  | 1.000 | 0.111 | 0.739 | 0.739 |
| LDL C   → PWPP | 1.932 | 5.597 | 0.002 | 0.004 |  | 1.080 | 0.764 | 0.354 | 0.700 |
| LpA   → PWPP | 1.000 | 0.039 | 0.844 | 0.844 |  | 1.000 | 0.663 | 0.416 | 0.416 |
| TG   → PWPP | 4.307 | 2.202 | 0.048 | 0.096 |  | 1.489 | 0.543 | 0.487 | 0.697 |
| TC   → PWPP | 1.961 | 4.332 | 0.010 | 0.019 |  | 1.777 | 0.943 | 0.347 | 0.562 |
| ApoA   → PR | 1.914 | 0.321 | 0.663 | 0.663 |  | 1.466 | 0.440 | 0.690 | 0.690 |
| ApoB   → PR | 4.497 | 1.720 | 0.132 | 0.132 |  | 2.020 | 2.032 | 0.135 | 0.161 |
| HDL C   → PR | 1.000 | 0.238 | 0.626 | 0.626 |  | 1.000 | 0.003 | 0.958 | 0.958 |
| LDL C   → PR | 1.616 | 0.981 | 0.372 | 0.372 |  | 2.067 | 1.629 | 0.206 | 0.206 |
| LpA   → PR | 7.046 | 1.535 | 0.127 | 0.253 |  | 1.000 | 1.216 | 0.270 | 0.477 |
| TG   → PR | 1.000 | 1.011 | 0.315 | 0.315 |  | 1.000 | 2.338 | 0.126 | 0.126 |
| TC   → PR | 1.476 | 0.963 | 0.476 | 0.476 |  | 1.837 | 1.037 | 0.350 | 0.350 |
| ASI   → ApoA | 1.000 | 5.118 | 0.024 | 0.048 |  | 1.515 | 0.866 | 0.337 | 0.426 |
| ASI   → ApoB | 1.000 | 0.639 | 0.424 | 0.424 |  | 1.000 | 0.313 | 0.576 | 0.576 |
| ASI   → HDL C | 1.000 | 2.402 | 0.122 | 0.244 |  | 1.000 | 1.162 | 0.281 | 0.562 |
| ASI   → LDL C | 1.000 | 0.052 | 0.820 | 0.820 |  | 1.000 | 0.586 | 0.444 | 0.500 |
| ASI   → LpA | 1.000 | 0.450 | 0.503 | 0.646 |  | 1.764 | 0.951 | 0.380 | 0.552 |
| ASI   → TG | 1.000 | 0.154 | 0.695 | 0.695 |  | 1.000 | 0.267 | 0.605 | 0.605 |
| ASI   → TC | 1.000 | 0.414 | 0.520 | 0.520 |  | 1.228 | 0.374 | 0.738 | 0.738 |
| PWRI   → ApoA | 1.000 | 0.092 | 0.762 | 0.762 |  | 1.000 | 0.133 | 0.715 | 0.781 |
| PWRI   → ApoB | 1.000 | 0.162 | 0.687 | 0.687 |  | 5.195 | 0.713 | 0.638 | 0.638 |
| PWRI   → HDL C | 1.000 | 0.500 | 0.480 | 0.480 |  | 1.000 | 0.010 | 0.920 | 0.964 |
| PWRI   → LDL C | 1.000 | 0.025 | 0.875 | 0.875 |  | 5.341 | 0.872 | 0.515 | 0.515 |
| PWRI   → LpA | 5.111 | 1.711 | 0.118 | 0.236 |  | 1.000 | 0.101 | 0.750 | 0.818 |
| PWRI   → TG | 1.000 | 0.733 | 0.392 | 0.785 |  | 1.000 | 3.717 | 0.054 | 0.108 |
| PWRI   → TC | 1.000 | 0.009 | 0.926 | 0.926 |  | 1.000 | 0.256 | 0.613 | 0.613 |
| PWPP   → ApoA | 1.000 | 6.068 | 0.014 | 0.028 |  | 5.741 | 1.385 | 0.203 | 0.405 |
| PWPP   → ApoB | 2.873 | 1.285 | 0.282 | 0.282 |  | 1.000 | 0.035 | 0.851 | 0.851 |
| PWPP   → HDL C | 1.000 | 2.298 | 0.130 | 0.261 |  | 5.470 | 0.789 | 0.566 | 0.739 |
| PWPP   → LDL C | 2.853 | 0.959 | 0.501 | 0.501 |  | 1.000 | 0.149 | 0.700 | 0.700 |
| PWPP   → LpA | 1.593 | 2.034 | 0.174 | 0.348 |  | 1.000 | 1.262 | 0.261 | 0.416 |
| PWPP   → TG | 1.000 | 0.911 | 0.340 | 0.340 |  | 1.684 | 0.287 | 0.697 | 0.697 |
| PWPP   → TC | 1.000 | 0.033 | 0.857 | 0.857 |  | 4.789 | 0.825 | 0.562 | 0.562 |
| PR   → ApoA | 2.152 | 1.292 | 0.379 | 0.663 |  | 1.000 | 1.125 | 0.289 | 0.578 |
| PR   → ApoB | 7.265 | 2.193 | 0.025 | 0.050 |  | 2.199 | 1.958 | 0.161 | 0.161 |
| PR   → HDL C | 2.266 | 1.225 | 0.374 | 0.626 |  | 2.539 | 0.715 | 0.503 | 0.958 |
| PR   → LDL C | 3.500 | 1.536 | 0.187 | 0.372 |  | 2.476 | 2.099 | 0.090 | 0.179 |
| PR   → LpA | 1.000 | 0.157 | 0.692 | 0.692 |  | 1.000 | 0.505 | 0.477 | 0.477 |
| PR   → TG | 5.805 | 1.733 | 0.101 | 0.202 |  | 1.000 | 2.892 | 0.089 | 0.126 |
| PR   → TC | 3.665 | 1.561 | 0.164 | 0.328 |  | 2.800 | 2.520 | 0.042 | 0.084 |
| Note: EDF indicates the effective degrees of freedom for the cross-lagged regression. Benjamini-Hochberg false discovery rate-corrected (FDR) P-value for multiple testing. Analyses were adjusted for Age, Smoking, Diabetes, SBP, Glucose, eGFR, CRP, IGF1, BMI, Albumin, ALP, ALT, AST, GGT, Phosphate, TBIL, TP, CysC, Urea, Urate, Testosterone, SHBG, Calcium and VitD at baseline. For categorical variables: the *p*-value was calculated using the Fisher exact test; for normally distributed continuous variables: the *p*-value was calculated using a t-test; for non-normally distributed continuous variables, the p-value was calculated using the Wilcoxon rank sum test.  Abbreviations: ASI, arterial stiffness index; PWRI, pulse wave reflection index; PWPP, pulse wave peak to peak time; PR, pulse rate; SBP, systolic blood pressure; TC, cholesterol; HDL-C, high-density lipoprotein cholesterol; LDL-C, low-density lipoprotein cholesterol; TG, triglycerides; LpA, lipoprotein A; ApoA, apolipoprotein A; ApoB, apolipoprotein B; BMI, Body mass index; ALP, alkaline phosphatase; ALT, alanine aminotransferase; AST, aspartate aminotransferase; GGT, gamma-glutamyltransferase; TBIL, total bilirubin; TP, total protein, eGFR, estimated glomerular filtration rate; CysC, cystatin C; SHBG, sex hormone binding globulin; CRP, c-reactive protein; VitD, vitamin D. | | | | | | | | | |

**Table S9. Formal Age-By-Variable Interaction Test for Directional Cross-Lagged Associations Between Lipid Measures and Arterial Stiffness Parameters**

| Association | EDF | F | *P* interaction | FDR *P* |
| --- | --- | --- | --- | --- |
| ApoA → ASI | 2.002 | 4.556 | 0.011 | 0.021 |
| ASI → ApoA | 2.001 | 0.573 | 0.565 | 0.565 |
| ApoB → ASI | 2.000 | 4.624 | 0.010 | 0.020 |
| ASI → ApoB | 2.003 | 1.550 | 0.213 | 0.213 |
| HDL_C → ASI | 2.002 | 4.821 | 0.008 | 0.016 |
| ASI → HDL_C | 2.003 | 0.852 | 0.426 | 0.426 |
| LDL_C → ASI | 2.001 | 4.628 | 0.010 | 0.020 |
| ASI → LDL_C | 2.006 | 1.773 | 0.171 | 0.171 |
| log(LpA) → ASI | 2.006 | 4.315 | 0.013 | 0.026 |
| ASI → log(LpA) | 3.251 | 1.710 | 0.268 | 0.268 |
| log(TG) → ASI | 2.002 | 4.381 | 0.013 | 0.013 |
| ASI → log(TG) | 2.001 | 10.988 | < 0.001 | < 0.001 |
| TC → ASI | 2.001 | 4.780 | 0.008 | 0.017 |
| ASI → TC | 2.003 | 0.889 | 0.412 | 0.412 |
| ApoA → log(PWRI) | 1.001 | 0.028 | 0.870 | 0.995 |
| log(PWRI) → ApoA | 1.000 | 0.000 | 0.995 | 0.995 |
| ApoB → log(PWRI) | 2.002 | 0.912 | 0.402 | 0.402 |
| log(PWRI) → ApoB | 1.001 | 1.165 | 0.281 | 0.402 |
| HDL_C → log(PWRI) | 1.001 | 0.198 | 0.657 | 0.657 |
| log(PWRI) → HDL_C | 1.001 | 0.260 | 0.610 | 0.657 |
| LDL_C → log(PWRI) | 1.001 | 0.224 | 0.637 | 0.637 |
| log(PWRI) → LDL_C | 1.002 | 0.820 | 0.365 | 0.637 |
| log(LpA) → log(PWRI) | 1.001 | 0.004 | 0.956 | 0.956 |
| log(PWRI) → log(LpA) | 1.001 | 1.353 | 0.245 | 0.490 |
| log(TG) → log(PWRI) | 2.143 | 0.765 | 0.483 | 0.483 |
| log(PWRI) → log(TG) | 2.001 | 10.576 | 0.000 | 0.000 |
| TC → log(PWRI) | 2.729 | 0.774 | 0.464 | 0.464 |
| log(PWRI) → TC | 1.005 | 0.853 | 0.357 | 0.464 |
| ApoA → log(PWPP) | 2.001 | 8.002 | < 0.001 | 0.001 |
| log(PWPP) → ApoA | 1.001 | 1.040 | 0.307 | 0.307 |
| ApoB → log(PWPP) | 1.001 | 0.031 | 0.862 | 0.862 |
| log(PWPP) → ApoB | 1.015 | 2.935 | 0.083 | 0.166 |
| HDL_C → log(PWPP) | 1.001 | 0.402 | 0.526 | 0.526 |
| log(PWPP) → HDL_C | 1.002 | 0.686 | 0.407 | 0.526 |
| LDL_C → log(PWPP) | 1.003 | 0.002 | 0.984 | 0.984 |
| log(PWPP) → LDL_C | 1.000 | 3.998 | 0.046 | 0.091 |
| log(LpA) → log(PWPP) | 1.086 | 0.067 | 0.924 | 0.924 |
| log(PWPP) → log(LpA) | 2.084 | 1.457 | 0.197 | 0.394 |
| log(TG) → log(PWPP) | 2.002 | 8.031 | < 0.001 | < 0.001 |
| log(PWPP) → log(TG) | 2.008 | 10.724 | < 0.001 | < 0.001 |
| TC→ log(PWPP) | 2.256 | 7.119 | 0.001 | 0.001 |
| log(PWPP) → TC | 1.001 | 1.671 | 0.196 | 0.196 |
| ApoA → PR | 1.003 | 0.074 | 0.788 | 0.971 |
| PR → ApoA | 1.000 | 0.002 | 0.971 | 0.971 |
| ApoB → PR | 2.089 | 2.017 | 0.196 | 0.197 |
| PR → ApoB | 1.010 | 1.640 | 0.197 | 0.197 |
| HDL_C → PR | 1.001 | 0.013 | 0.913 | 0.913 |
| PR → HDL_C | 1.002 | 0.057 | 0.814 | 0.913 |
| LDL_C → PR | 2.118 | 1.548 | 0.317 | 0.402 |
| PR → LDL_C | 1.003 | 0.702 | 0.402 | 0.402 |
| log(LpA) → PR | 2.001 | 0.512 | 0.600 | 0.647 |
| PR → log(LpA) | 1.001 | 0.210 | 0.647 | 0.647 |
| log(TG) → PR | 2.004 | 0.431 | 0.652 | 0.652 |
| PR → log(TG) | 2.003 | 12.052 | < 0.001 | < 0.001 |
| TC → PR | 2.751 | 0.458 | 0.660 | 0.660 |
| PR → TC | 1.000 | 0.552 | 0.457 | 0.660 |
| Note: EDF and F represent the estimated degrees of freedom and F statistic, respectively, for the age-by-variable interaction term. *P* interaction was obtained from the age-by-variable interaction term. Benjamini-Hochberg false discovery rate-corrected (FDR) *P*-value for multiple testing. Analyses were adjusted for Age, Smoking, Diabetes, SBP, Glucose, eGFR, CRP, IGF1, BMI, Albumin, ALP, ALT, AST, GGT, Phosphate, TBIL, TP, CysC, Urea, Urate, Testosterone, SHBG, Calcium and VitD at baseline. For categorical variables: the p-value was calculated using the Fisher exact test; for normally distributed continuous variables: the *p*-value was calculated using a t-test; for non-normally distributed continuous variables, the *p*-value was calculated using the Wilcoxon rank sum test.  Abbreviations: ASI, arterial stiffness index; PWRI, pulse wave reflection index; PWPP, pulse wave peak to peak time; PR, pulse rate; TC, cholesterol; HDL-C, high-density lipoprotein cholesterol; LDL-C, low-density lipoprotein cholesterol; TG, triglycerides; LpA, lipoprotein A; ApoA, apolipoprotein A; ApoB, apolipoprotein B; | | | | |

**Table S10. Directional Cross-Lagged Associations Between Lipid Measures and Arterial Stiffness Parameters in the Mixed Generalized Additive Cross-lagged Model.**

| Association | Male | | | |  | Female | | | |
| --- | --- | --- | --- | --- | --- | --- | --- | --- | --- |
|  | EDF | F | Raw *P* | FDR *P* |  | EDF | F | Raw *P* | FDR *P* |
| ASI → ApoA | 1.000 | 3.316 | 0.069 | 0.138 |  | 1.322 | 0.583 | 0.375 | 0.375 |
| ApoA → ASI | 1.000 | 0.043 | 0.836 | 0.836 |  | 2.110 | 1.986 | 0.175 | 0.351 |
| ASI → ApoB | 1.000 | 0.007 | 0.906 | 0.931 |  | 1.000 | 1.469 | 0.226 | 0.397 |
| ApoB → ASI | 1.000 | 0.310 | 0.578 | 0.931 |  | 2.260 | 0.770 | 0.397 | 0.397 |
| ASI → HDL-C | 1.769 | 2.162 | 0.096 | 0.224 |  | 1.317 | 0.423 | 0.453 | 0.528 |
| HDL-C → ASI | 1.220 | 0.120 | 0.720 | 0.749 |  | 1.024 | 0.425 | 0.528 | 0.528 |
| ASI → LDL-C | 1.000 | 0.175 | 0.657 | 0.676 |  | 1.000 | 1.181 | 0.277 | 0.277 |
| LDL-C → ASI | 1.000 | 0.467 | 0.494 | 0.676 |  | 1.000 | 1.775 | 0.183 | 0.277 |
| ASI → log(LpA) | 1.000 | 0.227 | 0.574 | 0.633 |  | 1.718 | 0.647 | 0.369 | 0.508 |
| log(LpA) → ASI | 2.064 | 0.724 | 0.358 | 0.633 |  | 1.000 | 0.439 | 0.508 | 0.508 |
| ASI → log(TG) | 1.000 | 1.674 | 0.172 | 0.392 |  | 1.000 | 0.025 | 0.876 | 0.876 |
| log(TG) → ASI | 1.217 | 0.670 | 0.510 | 0.568 |  | 2.800 | 5.162 | 0.004 | 0.009 |
| ASI → TC | 1.000 | 0.012 | 0.962 | 0.913 |  | 1.000 | 0.649 | 0.421 | 0.421 |
| TC → ASI | 1.000 | 0.279 | 0.597 | 0.913 |  | 1.000 | 1.394 | 0.238 | 0.421 |
| log(PWRI) → ApoA | 1.000 | 2.198 | 0.138 | 0.217 |  | 1.000 | 0.096 | 0.757 | 0.757 |
| ApoA → log(PWRI) | 1.000 | 1.524 | 0.217 | 0.217 |  | 1.809 | 0.645 | 0.492 | 0.757 |
| log(PWRI) → ApoB | 2.682 | 1.008 | 0.908 | 0.309 |  | 1.000 | 0.223 | 0.637 | 0.637 |
| ApoB → log(PWRI) | 2.780 | 3.352 | 0.047 | 0.038 |  | 1.000 | 2.219 | 0.137 | 0.273 |
| log(PWRI) → HDL-C | 1.000 | 2.168 | 0.138 | 0.209 |  | 1.000 | 0.213 | 0.644 | 0.897 |
| HDL-C → log(PWRI) | 1.000 | 1.578 | 0.212 | 0.209 |  | 1.000 | 0.017 | 0.897 | 0.897 |
| log(PWRI) →LDL-C | 2.683 | 0.922 | 0.985 | 0.365 |  | 1.000 | 0.001 | 0.981 | 0.981 |
| LDL-C → log(PWRI) | 2.788 | 2.801 | 0.123 | 0.089 |  | 1.000 | 3.302 | 0.069 | 0.139 |
| log(PWRI) → log(LpA) | 1.000 | 0.505 | 0.508 | 0.477 |  | 1.000 | 0.424 | 0.515 | 0.515 |
| log(LpA) → log(PWRI) | 1.675 | 1.708 | 0.194 | 0.353 |  | 1.000 | 2.235 | 0.135 | 0.270 |
| log(PWRI) → log(TG) | 1.000 | 0.394 | 0.520 | 0.530 |  | 1.000 | 0.003 | 0.959 | 0.959 |
| log(TG) → log(PWRI) | 1.436 | 2.299 | 0.168 | 0.360 |  | 1.000 | 8.042 | 0.005 | 0.009 |
| log(PWRI) → TC | 1.000 | 0.038 | 0.829 | 0.845 |  | 1.000 | 0.067 | 0.795 | 0.795 |
| TC → log(PWRI) | 2.826 | 3.000 | 0.097 | 0.066 |  | 1.000 | 3.893 | 0.049 | 0.097 |
| log(PWPP) → ApoA | 1.000 | 2.693 | 0.101 | 0.202 |  | 1.000 | 1.494 | 0.222 | 0.228 |
| ApoA → log(PWPP) | 1.000 | 0.017 | 0.897 | 0.897 |  | 1.977 | 1.417 | 0.228 | 0.228 |
| log(PWPP) → ApoB | 1.000 | 0.078 | 0.801 | 0.780 |  | 2.238 | 1.343 | 0.182 | 0.233 |
| ApoB → log(PWPP) | 1.000 | 0.932 | 0.334 | 0.669 |  | 2.423 | 1.171 | 0.233 | 0.233 |
| log(PWPP) → HDL-C | 1.854 | 1.665 | 0.146 | 0.395 |  | 1.003 | 1.401 | 0.237 | 0.475 |
| HDL-C → log(PWPP) | 1.233 | 0.093 | 0.912 | 0.900 |  | 1.261 | 0.175 | 0.857 | 0.857 |
| log(PWPP) → LDL-C | 1.000 | 0.000 | 0.987 | 0.995 |  | 2.597 | 1.627 | 0.117 | 0.145 |
| LDL-C → log(PWPP) | 1.000 | 0.943 | 0.332 | 0.663 |  | 1.000 | 2.128 | 0.145 | 0.145 |
| log(PWPP) → log(LpA) | 1.000 | 0.350 | 0.510 | 0.554 |  | 1.000 | 1.098 | 0.295 | 0.387 |
| log(LpA) → log(PWPP) | 1.610 | 0.630 | 0.512 | 0.554 |  | 1.000 | 0.747 | 0.387 | 0.387 |
| log(PWPP) → log(TG) | 1.000 | 0.816 | 0.336 | 0.706 |  | 1.000 | 0.005 | 0.946 | 0.946 |
| log(TG) → log(PWPP) | 1.321 | 0.461 | 0.673 | 0.706 |  | 2.797 | 4.577 | 0.006 | 0.011 |
| log(PWPP) → TC | 1.000 | 0.161 | 0.726 | 0.688 |  | 2.593 | 1.229 | 0.191 | 0.191 |
| TC → log(PWPP) | 1.000 | 0.717 | 0.397 | 0.688 |  | 1.000 | 1.987 | 0.159 | 0.191 |
| PR → ApoA | 1.811 | 1.478 | 0.231 | 0.522 |  | 1.000 | 0.018 | 0.894 | 0.894 |
| ApoA → PR | 1.385 | 0.340 | 0.539 | 0.560 |  | 1.000 | 0.589 | 0.443 | 0.886 |
| PR → ApoB | 1.000 | 0.312 | 0.553 | 0.577 |  | 1.947 | 1.250 | 0.248 | 0.451 |
| ApoB → PR | 2.377 | 0.992 | 0.341 | 0.566 |  | 1.000 | 0.568 | 0.451 | 0.451 |
| PR → HDL-C | 2.681 | 2.392 | 0.302 | 0.241 |  | 1.000 | 0.272 | 0.602 | 0.810 |
| HDL-C → PR | 1.000 | 0.264 | 0.607 | 0.607 |  | 1.000 | 0.058 | 0.810 | 0.810 |
| PR → LDL-C | 2.312 | 0.956 | 0.625 | 0.532 |  | 1.953 | 1.422 | 0.207 | 0.315 |
| LDL-C → PR | 2.284 | 0.937 | 0.282 | 0.532 |  | 2.501 | 0.911 | 0.315 | 0.315 |
| PR → log(LpA) | 1.000 | 0.038 | 0.875 | 0.846 |  | 1.000 | 0.288 | 0.592 | 0.644 |
| log(LpA) → PR | 1.563 | 0.638 | 0.455 | 0.846 |  | 1.000 | 0.213 | 0.644 | 0.644 |
| PR → log(TG) | 1.000 | 0.322 | 0.562 | 0.654 |  | 1.000 | 0.500 | 0.480 | 0.480 |
| log(TG) → PR | 1.498 | 0.387 | 0.661 | 0.654 |  | 1.000 | 6.152 | 0.013 | 0.026 |
| PR → TC | 2.644 | 1.807 | 0.486 | 0.342 |  | 2.077 | 2.029 | 0.148 | 0.296 |
| TC → PR | 2.278 | 0.789 | 0.351 | 0.342 |  | 1.000 | 1.072 | 0.301 | 0.301 |
| Note: EDF indicates the effective degrees of freedom for the cross-lagged regression. Benjamini-Hochberg false discovery rate-corrected (FDR) P-value for multiple testing. Analyses were adjusted for Age, Smoking, Diabetes, SBP, Glucose, eGFR, CRP, IGF1, BMI, Albumin, ALP, ALT, AST, GGT, Phosphate, TBIL, TP, CysC, Urea, Urate, Testosterone, SHBG, Calcium and VitD at baseline. For categorical variables: the p-value was calculated using the Fisher exact test; for normally distributed continuous variables: the p-value was calculated using a t-test; for non-normally distributed continuous variables, the p-value was calculated using the Wilcoxon rank sum test.  Abbreviations: ASI, arterial stiffness index; PWRI, pulse wave reflection index; PWPP, pulse wave peak to peak time; PR, pulse rate; TC, cholesterol; HDL-C, high-density lipoprotein cholesterol; LDL-C, low-density lipoprotein cholesterol; TG, triglycerides; LpA, lipoprotein A; ApoA, apolipoprotein A; ApoB, apolipoprotein B; | | | | | | | | | |

**Table S11. Directional Cross-Lagged Associations Between Lipid Measures and Arterial Stiffness Parameters in the Polynomial Regression-based Cross-lagged Model.**

| Association | Term | Male | | |  | Female | | |
| --- | --- | --- | --- | --- | --- | --- | --- | --- |
|  |  | *β* | Raw *P* | FDR *P* |  | *β* | Raw *P* | FDR *P* |
| ASI → ApoA | primary | -0.063 | 0.774 | 0.822 |  | -0.085 | 0.782 | 0.955 |
|  | quadratic | 0.097 | 0.822 | 0.822 |  | 0.049 | 0.936 | 0.955 |
|  | cubic | -0.067 | 0.766 | 0.822 |  | 0.019 | 0.955 | 0.955 |
| ApoA → ASI | primary | 0.474 | 0.620 | 0.822 |  | 1.745 | 0.230 | 0.675 |
|  | quadratic | -0.872 | 0.649 | 0.822 |  | -3.196 | 0.279 | 0.675 |
|  | cubic | 0.399 | 0.685 | 0.822 |  | 1.464 | 0.337 | 0.675 |
| ASI → ApoB | primary | -0.378 | 0.114 | 0.408 |  | 0.429 | 0.124 | 0.206 |
|  | quadratic | 0.682 | 0.145 | 0.408 |  | -0.771 | 0.171 | 0.206 |
|  | cubic | -0.314 | 0.204 | 0.408 |  | 0.376 | 0.215 | 0.215 |
| ApoB → ASI | primary | -0.247 | 0.728 | 0.728 |  | -1.106 | 0.162 | 0.206 |
|  | quadratic | 0.588 | 0.686 | 0.728 |  | 2.544 | 0.117 | 0.206 |
|  | cubic | -0.335 | 0.664 | 0.728 |  | -1.440 | 0.094 | 0.206 |
| ASI → HDL-C | primary | 0.074 | 0.713 | 0.919 |  | -0.058 | 0.826 | 0.985 |
|  | quadratic | -0.080 | 0.838 | 0.919 |  | 0.010 | 0.985 | 0.985 |
|  | cubic | -0.021 | 0.919 | 0.919 |  | 0.036 | 0.900 | 0.985 |
| HDL-C → ASI | primary | 0.619 | 0.310 | 0.621 |  | -0.032 | 0.960 | 0.985 |
|  | quadratic | -1.369 | 0.261 | 0.621 |  | 0.113 | 0.929 | 0.985 |
|  | cubic | 0.761 | 0.230 | 0.621 |  | -0.102 | 0.877 | 0.985 |
| ASI → LDL-C | primary | -0.332 | 0.170 | 0.255 |  | 0.510 | 0.071 | 0.213 |
|  | quadratic | 0.590 | 0.213 | 0.255 |  | -0.968 | 0.090 | 0.213 |
|  | cubic | -0.259 | 0.300 | 0.300 |  | 0.495 | 0.107 | 0.213 |
| LDL-C → ASI | primary | -0.987 | 0.107 | 0.214 |  | -0.464 | 0.513 | 0.513 |
|  | quadratic | 2.197 | 0.084 | 0.214 |  | 1.150 | 0.426 | 0.512 |
|  | cubic | -1.221 | 0.074 | 0.214 |  | -0.667 | 0.381 | 0.512 |
| ASI → log(LpA) | primary | -0.183 | 0.368 | 0.513 |  | -0.219 | 0.438 | 0.438 |
|  | quadratic | 0.309 | 0.438 | 0.513 |  | 0.586 | 0.306 | 0.438 |
|  | cubic | -0.137 | 0.513 | 0.513 |  | -0.369 | 0.231 | 0.438 |
| log(LpA) → ASI | primary | 1.067 | 0.174 | 0.513 |  | 0.665 | 0.395 | 0.438 |
|  | quadratic | -1.995 | 0.226 | 0.513 |  | -1.563 | 0.339 | 0.438 |
|  | cubic | 0.931 | 0.294 | 0.513 |  | 0.901 | 0.306 | 0.438 |
| ASI → log(TG) | primary | 0.076 | 0.785 | 0.805 |  | 0.118 | 0.734 | 0.761 |
|  | quadratic | -0.195 | 0.720 | 0.805 |  | -0.226 | 0.748 | 0.761 |
|  | cubic | 0.155 | 0.589 | 0.805 |  | 0.116 | 0.761 | 0.761 |
| log(TG) → ASI | primary | -0.012 | 0.805 | 0.805 |  | 0.037 | 0.321 | 0.642 |
|  | quadratic | 0.183 | 0.113 | 0.340 |  | 0.209 | 0.004 | 0.013 |
|  | cubic | -0.163 | 0.083 | 0.340 |  | -0.202 | 0.003 | 0.013 |
| ASI → TC | primary | -0.276 | 0.251 | 0.377 |  | 0.481 | 0.094 | 0.238 |
|  | quadratic | 0.465 | 0.323 | 0.388 |  | -0.936 | 0.107 | 0.238 |
|  | cubic | -0.197 | 0.427 | 0.427 |  | 0.487 | 0.119 | 0.238 |
| TC → ASI | primary | -1.834 | 0.030 | 0.060 |  | -0.528 | 0.560 | 0.560 |
|  | quadratic | 3.906 | 0.025 | 0.060 |  | 1.293 | 0.479 | 0.560 |
|  | cubic | -2.095 | 0.024 | 0.060 |  | -0.748 | 0.430 | 0.560 |
| log(PWRI) → ApoA | primary | 1.741 | 0.106 | 0.211 |  | 2.685 | 0.037 | 0.068 |
|  | quadratic | -3.509 | 0.094 | 0.211 |  | -4.865 | 0.039 | 0.068 |
|  | cubic | 1.783 | 0.088 | 0.211 |  | 2.208 | 0.043 | 0.068 |
| ApoA → log(PWRI) | primary | -0.242 | 0.802 | 0.802 |  | 2.805 | 0.055 | 0.068 |
|  | quadratic | 0.828 | 0.669 | 0.802 |  | -5.563 | 0.060 | 0.068 |
|  | cubic | -0.566 | 0.569 | 0.802 |  | 2.794 | 0.068 | 0.068 |
| log(PWRI) → ApoB | primary | -2.113 | 0.071 | 0.157 |  | -0.429 | 0.716 | 0.716 |
|  | quadratic | 3.967 | 0.082 | 0.157 |  | 0.846 | 0.695 | 0.716 |
|  | cubic | -1.892 | 0.096 | 0.157 |  | -0.414 | 0.677 | 0.716 |
| ApoB → log(PWRI) | primary | -1.040 | 0.148 | 0.157 |  | -0.901 | 0.256 | 0.512 |
|  | quadratic | 2.080 | 0.157 | 0.157 |  | 2.096 | 0.198 | 0.512 |
|  | cubic | -1.118 | 0.151 | 0.157 |  | -1.179 | 0.172 | 0.512 |
| log(PWRI) → HDL-C | primary | 1.722 | 0.079 | 0.158 |  | 1.395 | 0.207 | 0.414 |
|  | quadratic | -3.383 | 0.076 | 0.158 |  | -2.605 | 0.199 | 0.414 |
|  | cubic | 1.675 | 0.079 | 0.158 |  | 1.215 | 0.194 | 0.414 |
| HDL-C → log(PWRI) | primary | 0.448 | 0.467 | 0.641 |  | 0.486 | 0.445 | 0.445 |
|  | quadratic | -0.708 | 0.565 | 0.641 |  | -1.082 | 0.394 | 0.445 |
|  | cubic | 0.299 | 0.641 | 0.641 |  | 0.619 | 0.348 | 0.445 |
| log(PWRI) → LDL-C | primary | -1.835 | 0.121 | 0.149 |  | 0.132 | 0.912 | 0.951 |
|  | quadratic | 3.461 | 0.133 | 0.149 |  | -0.193 | 0.929 | 0.951 |
|  | cubic | -1.659 | 0.149 | 0.149 |  | 0.062 | 0.951 | 0.951 |
| LDL-C → log(PWRI) | primary | -0.961 | 0.121 | 0.149 |  | -0.610 | 0.391 | 0.782 |
|  | quadratic | 1.949 | 0.129 | 0.149 |  | 1.453 | 0.316 | 0.782 |
|  | cubic | -1.049 | 0.129 | 0.149 |  | -0.810 | 0.289 | 0.782 |
| log(PWRI) → log(LpA) | primary | 0.187 | 0.851 | 0.925 |  | -0.781 | 0.513 | 0.566 |
|  | quadratic | -0.353 | 0.855 | 0.925 |  | 1.420 | 0.516 | 0.566 |
|  | cubic | 0.158 | 0.870 | 0.925 |  | -0.634 | 0.530 | 0.566 |
| log(LpA) → log(PWRI) | primary | 0.075 | 0.925 | 0.925 |  | 0.455 | 0.562 | 0.566 |
|  | quadratic | 0.203 | 0.903 | 0.925 |  | -0.989 | 0.546 | 0.566 |
|  | cubic | -0.251 | 0.780 | 0.925 |  | 0.506 | 0.566 | 0.566 |
| log(PWRI) → log(TG) | primary | -1.585 | 0.245 | 0.493 |  | 3.151 | 0.032 | 0.063 |
|  | quadratic | 3.101 | 0.243 | 0.493 |  | -5.792 | 0.031 | 0.063 |
|  | cubic | -1.536 | 0.247 | 0.493 |  | 2.668 | 0.032 | 0.063 |
| log(TG) → log(PWRI) | primary | -0.008 | 0.873 | 0.984 |  | 0.055 | 0.142 | 0.142 |
|  | quadratic | 0.002 | 0.984 | 0.984 |  | 0.135 | 0.065 | 0.090 |
|  | cubic | -0.048 | 0.617 | 0.925 |  | -0.121 | 0.075 | 0.090 |
| log(PWRI) → TC | primary | -0.883 | 0.452 | 0.517 |  | 0.827 | 0.495 | 0.514 |
|  | quadratic | 1.606 | 0.483 | 0.517 |  | -1.493 | 0.501 | 0.514 |
|  | cubic | -0.741 | 0.517 | 0.517 |  | 0.668 | 0.514 | 0.514 |
| TC → log(PWRI) | primary | -1.453 | 0.090 | 0.197 |  | -1.201 | 0.186 | 0.372 |
|  | quadratic | 2.947 | 0.096 | 0.197 |  | 2.608 | 0.155 | 0.372 |
|  | cubic | -1.551 | 0.099 | 0.197 |  | -1.377 | 0.148 | 0.372 |
| log(PWPP) → ApoA | primary | 0.190 | 0.951 | 0.997 |  | -0.677 | 0.880 | 0.918 |
|  | quadratic | -0.153 | 0.979 | 0.997 |  | 1.154 | 0.897 | 0.918 |
|  | cubic | -0.010 | 0.997 | 0.997 |  | -0.455 | 0.918 | 0.918 |
| ApoA → log(PWPP) | primary | -0.320 | 0.738 | 0.997 |  | -1.891 | 0.195 | 0.556 |
|  | quadratic | 0.561 | 0.769 | 0.997 |  | 3.527 | 0.234 | 0.556 |
|  | cubic | -0.248 | 0.801 | 0.997 |  | -1.663 | 0.278 | 0.556 |
| log(PWPP) → ApoB | primary | -1.740 | 0.602 | 0.997 |  | -3.292 | 0.420 | 0.420 |
|  | quadratic | 2.963 | 0.646 | 0.997 |  | 7.052 | 0.383 | 0.420 |
|  | cubic | -1.222 | 0.697 | 0.997 |  | -3.794 | 0.346 | 0.420 |
| ApoB → log(PWPP) | primary | -0.064 | 0.928 | 0.997 |  | 1.027 | 0.195 | 0.390 |
|  | quadratic | 0.038 | 0.979 | 0.997 |  | -2.473 | 0.129 | 0.386 |
|  | cubic | 0.003 | 0.997 | 0.997 |  | 1.443 | 0.094 | 0.386 |
| log(PWPP) → HDL-C | primary | 2.014 | 0.472 | 0.583 |  | -1.195 | 0.756 | 0.940 |
|  | quadratic | -3.438 | 0.525 | 0.583 |  | 2.203 | 0.772 | 0.940 |
|  | cubic | 1.444 | 0.583 | 0.583 |  | -0.991 | 0.794 | 0.940 |
| HDL-C → log(PWPP) | primary | -0.568 | 0.352 | 0.583 |  | 0.048 | 0.940 | 0.940 |
|  | quadratic | 1.221 | 0.316 | 0.583 |  | -0.176 | 0.890 | 0.940 |
|  | cubic | -0.674 | 0.288 | 0.583 |  | 0.140 | 0.833 | 0.940 |
| log(PWPP) → LDL-C | primary | -2.726 | 0.420 | 0.501 |  | -6.514 | 0.115 | 0.231 |
|  | quadratic | 4.857 | 0.457 | 0.501 |  | 13.422 | 0.101 | 0.231 |
|  | cubic | -2.138 | 0.501 | 0.501 |  | -6.947 | 0.088 | 0.231 |
| LDL-C → log(PWPP) | primary | 0.846 | 0.167 | 0.334 |  | 0.392 | 0.582 | 0.582 |
|  | quadratic | -1.907 | 0.133 | 0.334 |  | -1.067 | 0.462 | 0.554 |
|  | cubic | 1.062 | 0.120 | 0.334 |  | 0.652 | 0.393 | 0.554 |
| log(PWPP) → log(LpA) | primary | -1.276 | 0.654 | 0.671 |  | 7.159 | 0.085 | 0.172 |
|  | quadratic | 2.416 | 0.661 | 0.671 |  | -14.176 | 0.085 | 0.172 |
|  | cubic | -1.135 | 0.671 | 0.671 |  | 7.015 | 0.086 | 0.172 |
| log(LpA) → log(PWPP) | primary | -0.806 | 0.304 | 0.671 |  | -0.788 | 0.315 | 0.315 |
|  | quadratic | 1.508 | 0.359 | 0.671 |  | 1.851 | 0.259 | 0.311 |
|  | cubic | -0.696 | 0.433 | 0.671 |  | -1.065 | 0.228 | 0.311 |
| log(PWPP) → log(TG) | primary | -2.047 | 0.600 | 0.795 |  | 1.575 | 0.758 | 0.759 |
|  | quadratic | 3.621 | 0.631 | 0.795 |  | -3.115 | 0.758 | 0.759 |
|  | cubic | -1.598 | 0.663 | 0.795 |  | 1.542 | 0.759 | 0.759 |
| log(TG) → log(PWPP) | primary | 0.009 | 0.853 | 0.853 |  | -0.044 | 0.239 | 0.477 |
|  | quadratic | -0.166 | 0.150 | 0.450 |  | -0.181 | 0.014 | 0.041 |
|  | cubic | 0.156 | 0.099 | 0.450 |  | 0.187 | 0.006 | 0.037 |
| log(PWPP) → TC | primary | -2.671 | 0.426 | 0.484 |  | -5.721 | 0.174 | 0.349 |
|  | quadratic | 4.878 | 0.452 | 0.484 |  | 11.810 | 0.157 | 0.349 |
|  | cubic | -2.207 | 0.484 | 0.484 |  | -6.119 | 0.140 | 0.349 |
| TC → log(PWPP) | primary | 1.716 | 0.042 | 0.085 |  | 0.522 | 0.566 | 0.566 |
|  | quadratic | -3.693 | 0.035 | 0.085 |  | -1.354 | 0.460 | 0.552 |
|  | cubic | 1.990 | 0.032 | 0.085 |  | 0.811 | 0.394 | 0.552 |
| PR → ApoA | primary | 0.360 | 0.552 | 0.820 |  | 0.302 | 0.763 | 0.795 |
|  | quadratic | -0.478 | 0.686 | 0.820 |  | -0.555 | 0.779 | 0.795 |
|  | cubic | 0.135 | 0.820 | 0.820 |  | 0.258 | 0.795 | 0.795 |
| ApoA → PR | primary | -0.753 | 0.330 | 0.820 |  | -0.489 | 0.682 | 0.795 |
|  | quadratic | 1.188 | 0.442 | 0.820 |  | 1.030 | 0.671 | 0.795 |
|  | cubic | -0.455 | 0.567 | 0.820 |  | -0.530 | 0.672 | 0.795 |
| PR → ApoB | primary | 0.752 | 0.253 | 0.442 |  | 0.022 | 0.981 | 0.981 |
|  | quadratic | -1.519 | 0.237 | 0.442 |  | 0.269 | 0.881 | 0.981 |
|  | cubic | 0.793 | 0.219 | 0.442 |  | -0.287 | 0.752 | 0.981 |
| ApoB → PR | primary | -0.351 | 0.542 | 0.542 |  | 0.744 | 0.251 | 0.503 |
|  | quadratic | 1.007 | 0.392 | 0.470 |  | -1.645 | 0.217 | 0.503 |
|  | cubic | -0.652 | 0.294 | 0.442 |  | 0.905 | 0.200 | 0.503 |
| PR → HDL-C | primary | 0.542 | 0.326 | 0.906 |  | 0.653 | 0.447 | 0.621 |
|  | quadratic | -0.934 | 0.386 | 0.906 |  | -1.295 | 0.445 | 0.621 |
|  | cubic | 0.406 | 0.453 | 0.906 |  | 0.659 | 0.440 | 0.621 |
| HDL-C → PR | primary | -0.207 | 0.673 | 0.988 |  | 0.413 | 0.427 | 0.621 |
|  | quadratic | 0.207 | 0.833 | 0.988 |  | -0.669 | 0.519 | 0.621 |
|  | cubic | -0.008 | 0.988 | 0.988 |  | 0.267 | 0.621 | 0.621 |
| PR → LDL-C | primary | 0.795 | 0.233 | 0.466 |  | 0.254 | 0.784 | 0.965 |
|  | quadratic | -1.548 | 0.233 | 0.466 |  | -0.206 | 0.910 | 0.965 |
|  | cubic | 0.780 | 0.232 | 0.466 |  | -0.040 | 0.965 | 0.965 |
| LDL-C → PR | primary | -0.089 | 0.857 | 0.857 |  | 0.889 | 0.126 | 0.252 |
|  | quadratic | 0.470 | 0.648 | 0.777 |  | -1.995 | 0.092 | 0.252 |
|  | cubic | -0.381 | 0.491 | 0.736 |  | 1.112 | 0.075 | 0.252 |
| PR → log(LpA) | primary | -0.866 | 0.122 | 0.328 |  | 0.104 | 0.911 | 0.994 |
|  | quadratic | 1.618 | 0.139 | 0.328 |  | -0.102 | 0.956 | 0.994 |
|  | cubic | -0.763 | 0.164 | 0.328 |  | 0.007 | 0.994 | 0.994 |
| log(LpA) → PR | primary | -0.044 | 0.945 | 0.945 |  | 0.398 | 0.535 | 0.994 |
|  | quadratic | 0.239 | 0.857 | 0.945 |  | -0.940 | 0.483 | 0.994 |
|  | cubic | -0.218 | 0.762 | 0.945 |  | 0.545 | 0.449 | 0.994 |
| PR → log(TG) | primary | -0.245 | 0.750 | 0.900 |  | -0.553 | 0.628 | 0.662 |
|  | quadratic | 0.497 | 0.740 | 0.900 |  | 1.188 | 0.598 | 0.662 |
|  | cubic | -0.270 | 0.720 | 0.900 |  | -0.659 | 0.561 | 0.662 |
| log(TG) → PR | primary | 0.031 | 0.435 | 0.900 |  | -0.048 | 0.114 | 0.662 |
|  | quadratic | -0.039 | 0.674 | 0.900 |  | 0.026 | 0.662 | 0.662 |
|  | cubic | 0.004 | 0.954 | 0.954 |  | -0.035 | 0.532 | 0.662 |
| PR → TC | primary | 0.974 | 0.141 | 0.306 |  | 0.511 | 0.588 | 0.850 |
|  | quadratic | -1.875 | 0.146 | 0.306 |  | -0.677 | 0.716 | 0.850 |
|  | cubic | 0.925 | 0.153 | 0.306 |  | 0.176 | 0.850 | 0.850 |
| TC → PR | primary | -0.423 | 0.536 | 0.536 |  | 0.526 | 0.478 | 0.850 |
|  | quadratic | 1.177 | 0.405 | 0.486 |  | -1.270 | 0.396 | 0.850 |
|  | cubic | -0.760 | 0.311 | 0.467 |  | 0.736 | 0.344 | 0.850 |
| Note: β indicates the effect size for the cross-lagged regression. Benjamini-Hochberg false discovery rate-corrected (FDR) P-value for multiple testing. Analyses were adjusted for Age, Smoking, Diabetes, SBP, Glucose, eGFR, CRP, IGF1, BMI, Albumin, ALP, ALT, AST, GGT, Phosphate, TBIL, TP, CysC, Urea, Urate, Testosterone, SHBG, Calcium and VitD at baseline. For categorical variables: the p-value was calculated using the Fisher exact test; for normally distributed continuous variables: the p-value was calculated using a t-test; for non-normally distributed continuous variables, the p-value was calculated using the Wilcoxon rank sum test.  Abbreviations: Abbreviations: ASI, arterial stiffness index; PWRI, pulse wave reflection index; PWPP, pulse wave peak to peak time; PR, pulse rate; TC, cholesterol; HDL-C, high-density lipoprotein cholesterol; LDL-C, low-density lipoprotein cholesterol; TG, triglycerides; LpA, lipoprotein A; ApoA, apolipoprotein A; ApoB, apolipoprotein B; | | | | | | | | |

**Table S12. Sensitivity Analysis of GACM and Mixed-GACM Using Untransformed Variables**

|  | GACM | | | | | | | | |  | | Mixed-GACM | | | | | | | | | |
| --- | --- | --- | --- | --- | --- | --- | --- | --- | --- | --- | --- | --- | --- | --- | --- | --- | --- | --- | --- | --- | --- |
| Association | Male | | | |  | Female | | | | |  | | Male | | | |  | Female | | | |
|  | EDF | F | Raw *P* | FDR *P* |  | EDF | F | Raw *P* | FDR *P* | |  | | EDF | F | Raw *P* | FDR *P* |  | EDF | F | Raw *P* | FDR *P* |
| ASI → ApoA | 1.000 | 4.344 | 0.037 | 0.075 |  | 1.000 | 1.327 | 0.250 | 0.499 | |  | | 1.000 | 4.297 | 0.038 | 0.077 |  | 1.000 | 1.327 | 0.250 | 0.476 |
| ApoA → ASI | 4.448 | 0.754 | 0.615 | 0.615 |  | 1.672 | 0.691 | 0.535 | 0.535 | |  | | 1.000 | 0.018 | 0.893 | 0.893 |  | 1.758 | 0.609 | 0.476 | 0.476 |
| ASI → ApoB | 1.418 | 0.470 | 0.697 | 0.697 |  | 1.342 | 0.473 | 0.477 | 0.477 | |  | | 1.452 | 0.541 | 0.672 | 0.672 |  | 1.290 | 0.527 | 0.408 | 0.408 |
| ApoB → ASI | 1.000 | 0.205 | 0.651 | 0.697 |  | 1.003 | 1.661 | 0.197 | 0.393 | |  | | 1.000 | 0.205 | 0.651 | 0.672 |  | 1.043 | 1.554 | 0.203 | 0.405 |
| ASI → HDL-C | 1.000 | 4.010 | 0.045 | 0.091 |  | 1.000 | 1.123 | 0.290 | 0.579 | |  | | 1.000 | 3.875 | 0.049 | 0.098 |  | 1.000 | 0.902 | 0.342 | 0.651 |
| HDL-C → ASI | 1.000 | 0.161 | 0.689 | 0.689 |  | 1.000 | 0.205 | 0.651 | 0.651 | |  | | 1.000 | 0.161 | 0.689 | 0.689 |  | 1.000 | 0.205 | 0.651 | 0.651 |
| ASI → LDL-C | 1.436 | 0.814 | 0.538 | 0.565 |  | 4.044 | 1.035 | 0.406 | 0.406 | |  | | 1.516 | 1.004 | 0.496 | 0.565 |  | 1.000 | 0.904 | 0.342 | 0.342 |
| LDL-C → ASI | 1.000 | 0.332 | 0.565 | 0.565 |  | 1.000 | 2.053 | 0.152 | 0.304 | |  | | 1.000 | 0.332 | 0.565 | 0.565 |  | 1.000 | 2.053 | 0.152 | 0.304 |
| ASI → LpA | 1.000 | 1.154 | 0.283 | 0.303 |  | 1.054 | 2.753 | 0.084 | 0.167 | |  | | 1.000 | 1.292 | 0.256 | 0.303 |  | 1.000 | 3.330 | 0.068 | 0.137 |
| LpA → ASI | 1.000 | 1.060 | 0.303 | 0.303 |  | 1.002 | 0.145 | 0.704 | 0.704 | |  | | 1.000 | 1.060 | 0.303 | 0.303 |  | 1.015 | 0.137 | 0.710 | 0.710 |
| ASI → TG | 1.000 | 2.913 | 0.088 | 0.176 |  | 1.001 | 1.142 | 0.285 | 0.285 | |  | | 1.000 | 3.293 | 0.070 | 0.140 |  | 1.000 | 1.188 | 0.276 | 0.276 |
| TG → ASI | 2.775 | 0.829 | 0.416 | 0.416 |  | 6.693 | 2.038 | 0.041 | 0.081 | |  | | 1.761 | 0.525 | 0.468 | 0.468 |  | 2.167 | 3.121 | 0.032 | 0.064 |
| ASI → TC | 1.481 | 0.376 | 0.718 | 0.718 |  | 1.000 | 0.497 | 0.481 | 0.481 | |  | | 1.453 | 0.345 | 0.749 | 0.749 |  | 1.000 | 0.555 | 0.457 | 0.457 |
| TC → ASI | 2.992 | 0.724 | 0.528 | 0.718 |  | 1.000 | 1.856 | 0.173 | 0.347 | |  | | 1.000 | 0.185 | 0.668 | 0.749 |  | 1.000 | 1.856 | 0.173 | 0.347 |
| PWRI → ApoA | 1.662 | 0.818 | 0.468 | 0.879 |  | 5.059 | 1.004 | 0.420 | 0.700 | |  | | 1.660 | 0.462 | 0.482 | 0.879 |  | 1.000 | 0.602 | 0.438 | 0.694 |
| ApoA → PWRI | 1.000 | 0.023 | 0.879 | 0.879 |  | 1.000 | 0.149 | 0.700 | 0.700 | |  | | 1.000 | 0.023 | 0.879 | 0.879 |  | 1.000 | 0.155 | 0.694 | 0.694 |
| PWRI → ApoB | 5.590 | 0.592 | 0.714 | 0.714 |  | 1.000 | 0.070 | 0.791 | 0.791 | |  | | 1.000 | 0.397 | 0.529 | 0.529 |  | 1.000 | 0.070 | 0.791 | 0.791 |
| ApoB → PWRI | 1.252 | 4.229 | 0.019 | 0.039 |  | 1.000 | 0.101 | 0.751 | 0.791 | |  | | 1.297 | 4.762 | 0.016 | 0.031 |  | 1.000 | 0.097 | 0.756 | 0.791 |
| PWRI → HDL-C | 1.178 | 0.398 | 0.506 | 0.961 |  | 1.000 | 0.258 | 0.612 | 0.739 | |  | | 1.251 | 0.408 | 0.473 | 0.946 |  | 1.000 | 0.296 | 0.587 | 0.735 |
| HDL-C → PWRI | 1.000 | 0.002 | 0.961 | 0.961 |  | 1.000 | 0.111 | 0.739 | 0.739 | |  | | 1.000 | 0.002 | 0.961 | 0.961 |  | 1.000 | 0.115 | 0.735 | 0.735 |
| PWRI → LDL-C | 1.000 | 0.211 | 0.646 | 0.646 |  | 1.000 | 0.015 | 0.903 | 0.903 | |  | | 1.000 | 0.210 | 0.647 | 0.647 |  | 1.000 | 0.012 | 0.913 | 0.913 |
| LDL-C → PWRI | 1.000 | 4.861 | 0.028 | 0.055 |  | 1.000 | 0.287 | 0.592 | 0.903 | |  | | 1.000 | 4.861 | 0.028 | 0.055 |  | 1.000 | 0.283 | 0.595 | 0.913 |
| PWRI → LpA | 1.002 | 0.234 | 0.629 | 0.629 |  | 1.001 | 0.227 | 0.634 | 0.634 | |  | | 1.000 | 0.256 | 0.613 | 0.613 |  | 1.000 | 0.236 | 0.627 | 0.627 |
| LpA → PWRI | 2.881 | 1.454 | 0.184 | 0.368 |  | 1.000 | 0.789 | 0.374 | 0.634 | |  | | 2.174 | 2.205 | 0.174 | 0.349 |  | 1.000 | 0.784 | 0.376 | 0.627 |
| PWRI → TG | 5.683 | 1.131 | 0.320 | 0.422 |  | 3.501 | 0.981 | 0.439 | 0.439 | |  | | 1.000 | 0.404 | 0.525 | 0.525 |  | 1.051 | 0.089 | 0.832 | 0.832 |
| TG → PWRI | 1.000 | 0.646 | 0.422 | 0.422 |  | 1.915 | 2.738 | 0.047 | 0.095 | |  | | 1.000 | 0.646 | 0.422 | 0.525 |  | 1.555 | 3.265 | 0.032 | 0.064 |
| PWRI → TC | 1.000 | 0.153 | 0.696 | 0.696 |  | 1.002 | 0.001 | 0.989 | 0.989 | |  | | 1.000 | 0.151 | 0.697 | 0.697 |  | 1.000 | < 0.001 | 0.996 | 0.996 |
| TC → PWRI | 1.000 | 4.061 | 0.044 | 0.088 |  | 1.000 | 0.406 | 0.524 | 0.989 | |  | | 1.000 | 4.061 | 0.044 | 0.088 |  | 1.000 | 0.400 | 0.527 | 0.996 |
| PWPP → ApoA | 1.393 | 1.557 | 0.149 | 0.297 |  | 1.000 | 2.242 | 0.135 | 0.269 | |  | | 1.401 | 1.755 | 0.121 | 0.242 |  | 1.000 | 2.242 | 0.135 | 0.269 |
| ApoA → PWPP | 4.205 | 0.625 | 0.750 | 0.750 |  | 1.000 | 0.460 | 0.498 | 0.498 | |  | | 1.000 | 0.067 | 0.796 | 0.796 |  | 1.000 | 0.460 | 0.498 | 0.498 |
| PWPP → ApoB | 1.000 | 0.074 | 0.786 | 0.786 |  | 1.535 | 1.573 | 0.162 | 0.162 | |  | | 1.000 | 0.071 | 0.790 | 0.790 |  | 1.621 | 2.872 | 0.149 | 0.149 |
| ApoB → PWPP | 1.000 | 2.140 | 0.144 | 0.288 |  | 2.124 | 1.730 | 0.128 | 0.162 | |  | | 1.000 | 2.140 | 0.144 | 0.288 |  | 2.046 | 2.373 | 0.080 | 0.149 |
| PWPP → HDL-C | 1.736 | 1.169 | 0.342 | 0.684 |  | 1.000 | 1.845 | 0.175 | 0.349 | |  | | 1.706 | 0.704 | 0.339 | 0.678 |  | 1.000 | 1.649 | 0.199 | 0.399 |
| HDL-C → PWPP | 1.000 | 0.005 | 0.945 | 0.945 |  | 1.000 | 0.013 | 0.911 | 0.911 | |  | | 1.000 | 0.005 | 0.945 | 0.945 |  | 1.000 | 0.013 | 0.911 | 0.911 |
| PWPP → LDL-C | 2.787 | 0.653 | 0.601 | 0.601 |  | 1.979 | 2.048 | 0.122 | 0.174 | |  | | 1.000 | < 0.001 | 0.988 | 0.988 |  | 1.848 | 3.148 | 0.105 | 0.159 |
| LDL-C → PWPP | 1.000 | 1.689 | 0.194 | 0.388 |  | 1.735 | 1.654 | 0.174 | 0.174 | |  | | 1.000 | 1.689 | 0.194 | 0.388 |  | 1.654 | 1.369 | 0.159 | 0.159 |
| PWPP → LpA | 1.001 | 0.560 | 0.454 | 0.454 |  | 1.021 | 2.932 | 0.088 | 0.177 | |  | | 1.000 | 0.609 | 0.435 | 0.435 |  | 1.000 | 3.091 | 0.079 | 0.158 |
| LpA → PWPP | 1.000 | 0.775 | 0.379 | 0.454 |  | 1.540 | 0.427 | 0.592 | 0.592 | |  | | 1.000 | 0.775 | 0.379 | 0.435 |  | 1.547 | 0.273 | 0.610 | 0.610 |
| PWPP → TG | 1.442 | 0.717 | 0.367 | 0.733 |  | 1.001 | 0.756 | 0.385 | 0.385 | |  | | 1.273 | 0.593 | 0.358 | 0.716 |  | 1.000 | 0.784 | 0.376 | 0.376 |
| TG → PWPP | 1.000 | 0.106 | 0.745 | 0.745 |  | 7.470 | 1.754 | 0.123 | 0.246 | |  | | 1.000 | 0.106 | 0.745 | 0.745 |  | 1.964 | 1.550 | 0.172 | 0.345 |
| PWPP → TC | 1.000 | 0.110 | 0.740 | 0.740 |  | 2.538 | 1.433 | 0.258 | 0.258 | |  | | 1.000 | 0.108 | 0.742 | 0.742 |  | 1.863 | 1.961 | 0.226 | 0.226 |
| TC → PWPP | 1.117 | 0.834 | 0.334 | 0.668 |  | 3.842 | 1.631 | 0.128 | 0.256 | |  | | 1.104 | 0.960 | 0.303 | 0.607 |  | 1.749 | 1.473 | 0.146 | 0.226 |
| PR → ApoA | 1.718 | 0.734 | 0.512 | 0.512 |  | 1.000 | 0.263 | 0.608 | 0.989 | |  | | 1.715 | 0.583 | 0.456 | 0.456 |  | 1.000 | 0.263 | 0.608 | 0.976 |
| ApoA → PR | 2.099 | 1.458 | 0.274 | 0.512 |  | 1.000 | < 0.001 | 0.989 | 0.989 | |  | | 1.898 | 1.310 | 0.197 | 0.394 |  | 1.000 | 0.001 | 0.976 | 0.976 |
| PR → ApoB | 1.000 | 0.004 | 0.949 | 0.949 |  | 1.868 | 1.144 | 0.294 | 0.588 | |  | | 1.000 | 0.002 | 0.965 | 0.965 |  | 1.948 | 1.510 | 0.188 | 0.375 |
| ApoB → PR | 6.490 | 1.550 | 0.138 | 0.275 |  | 1.000 | 0.194 | 0.660 | 0.660 | |  | | 1.854 | 0.917 | 0.297 | 0.594 |  | 1.000 | 0.203 | 0.653 | 0.653 |
| PR → HDL-C | 1.000 | 0.053 | 0.818 | 0.818 |  | 1.000 | 0.044 | 0.834 | 0.834 | |  | | 1.033 | 0.074 | 0.834 | 0.834 |  | 1.000 | 0.058 | 0.810 | 0.810 |
| HDL-C → PR | 4.248 | 1.211 | 0.347 | 0.693 |  | 1.000 | 0.471 | 0.493 | 0.834 | |  | | 1.628 | 0.489 | 0.432 | 0.834 |  | 1.000 | 0.524 | 0.469 | 0.810 |
| PR → LDL-C | 1.000 | 0.018 | 0.894 | 0.894 |  | 1.772 | 1.076 | 0.342 | 0.501 | |  | | 1.000 | 0.017 | 0.897 | 0.897 |  | 1.836 | 1.006 | 0.278 | 0.556 |
| LDL-C → PR | 4.778 | 1.349 | 0.270 | 0.539 |  | 2.534 | 0.858 | 0.501 | 0.501 | |  | | 1.993 | 1.086 | 0.317 | 0.635 |  | 1.971 | 0.343 | 0.684 | 0.684 |
| PR → LpA | 4.782 | 0.928 | 0.462 | 0.462 |  | 1.000 | 0.022 | 0.882 | 0.882 | |  | | 1.000 | 0.120 | 0.729 | 0.729 |  | 1.000 | 0.023 | 0.879 | 0.879 |
| LpA → PR | 1.000 | 1.267 | 0.260 | 0.462 |  | 1.000 | 0.093 | 0.760 | 0.882 | |  | | 1.000 | 1.267 | 0.260 | 0.521 |  | 1.000 | 0.109 | 0.742 | 0.879 |
| PR → TG | 1.001 | 1.116 | 0.291 | 0.582 |  | 3.220 | 1.504 | 0.214 | 0.214 | |  | | 1.000 | 1.188 | 0.276 | 0.552 |  | 1.506 | 0.418 | 0.467 | 0.467 |
| TG → PR | 1.078 | 0.253 | 0.728 | 0.728 |  | 1.000 | 2.892 | 0.089 | 0.178 | |  | | 1.109 | 0.265 | 0.707 | 0.707 |  | 1.000 | 2.839 | 0.092 | 0.184 |
| PR → TC | 1.000 | 0.301 | 0.583 | 0.583 |  | 1.979 | 1.313 | 0.255 | 0.297 | |  | | 1.000 | 0.299 | 0.585 | 0.585 |  | 2.000 | 1.838 | 0.159 | 0.279 |
| TC → PR | 3.132 | 1.114 | 0.343 | 0.583 |  | 1.000 | 1.086 | 0.297 | 0.297 | |  | | 2.203 | 0.638 | 0.393 | 0.585 |  | 1.000 | 1.174 | 0.279 | 0.279 |
| Note: EDF indicates the effective degrees of freedom for the cross-lagged regression. Benjamini-Hochberg false discovery rate-corrected (FDR) P-values for multiple testing. *β* and their corresponding 95% confidence intervals (CIs) were reported alongside raw P-values and FDR P-values. Analyses were adjusted for Age, Smoking, Diabetes, SBP, Glucose, eGFR, CRP, IGF1, BMI, Albumin, ALP, ALT, AST, GGT, Phosphate, TBIL, TP, CysC, Urea, Urate, Testosterone, SHBG, Calcium and VitD at baseline. For categorical variables: the p-value was calculated using the Fisher exact test; for normally distributed continuous variables: the p-value was calculated using a t-test; for non-normally distributed continuous variables, the p-value was calculated using the Wilcoxon rank sum test. | | | | | | | | | | | | | | | | | | | | | |
| Abbreviations: Abbreviations: ASI, arterial stiffness index; PWRI, pulse wave reflection index; PWPP, pulse wave peak to peak time; PR, pulse rate; TC, Cholesterol; HDL-C, high-density lipoprotein Cholesterol; LDL-C, low-density lipoprotein Cholesterol; TG, triglycerides; LpA, lipoprotein A; ApoA, apolipoprotein A; ApoB, apolipoprotein B; | | | | | | | | | | | | | | | | | | | | | |

**Table S13. Directional Cross-Lagged Associations Between Lipid Measures and Arterial Stiffness Parameters in the Generalized Additive Cross-lagged Model Incorporating SBP as a Third Endogenous Variable**

| Association | Male | | | |  | Female | | | |
| --- | --- | --- | --- | --- | --- | --- | --- | --- | --- |
|  | EDF | F | Raw *P* | FDR *P* |  | EDF | F | Raw *P* | FDR *P* |
| ASI → ApoA | 1.000 | 3.883 | 0.049 | 0.098 |  | 1.376 | 0.525 | 0.441 | 0.441 |
| ApoA → ASI | 1.000 | 0.052 | 0.820 | 0.820 |  | 5.683 | 1.647 | 0.118 | 0.236 |
| ASI → ApoB | 1.634 | 0.509 | 0.614 | 0.878 |  | 1.000 | 1.686 | 0.194 | 0.375 |
| ApoB → ASI | 1.000 | 0.023 | 0.878 | 0.878 |  | 2.864 | 1.041 | 0.375 | 0.375 |
| ASI → HDL-C | 4.082 | 1.901 | 0.091 | 0.183 |  | 1.339 | 0.392 | 0.514 | 0.615 |
| HDL-C → ASI | 1.000 | 0.302 | 0.583 | 0.583 |  | 1.472 | 0.616 | 0.615 | 0.615 |
| ASI → LDL-C | 1.576 | 0.559 | 0.591 | 0.654 |  | 4.443 | 1.161 | 0.356 | 0.356 |
| LDL-C → ASI | 2.977 | 0.713 | 0.654 | 0.654 |  | 1.000 | 1.754 | 0.186 | 0.356 |
| ASI → log(LpA) | 1.000 | 0.906 | 0.341 | 0.341 |  | 2.261 | 2.693 | 0.040 | 0.080 |
| log(LpA) → ASI | 1.000 | 1.600 | 0.206 | 0.341 |  | 1.000 | 0.223 | 0.637 | 0.637 |
| ASI → log(TG) | 1.000 | 0.214 | 0.643 | 0.643 |  | 1.000 | 0.006 | 0.941 | 0.941 |
| log(TG) → ASI | 6.990 | 1.330 | 0.226 | 0.451 |  | 2.655 | 2.245 | 0.066 | 0.132 |
| ASI → TC | 1.545 | 0.330 | 0.711 | 0.711 |  | 4.254 | 1.077 | 0.382 | 0.382 |
| TC → ASI | 2.834 | 0.696 | 0.619 | 0.711 |  | 1.000 | 1.314 | 0.252 | 0.382 |
| log(PWRI) → ApoA | 1.788 | 1.427 | 0.244 | 0.488 |  | 3.938 | 1.326 | 0.250 | 0.500 |
| ApoA → log(PWRI) | 1.000 | 0.284 | 0.594 | 0.594 |  | 1.492 | 0.500 | 0.504 | 0.504 |
| log(PWRI) → ApoB | 4.154 | 1.469 | 0.247 | 0.494 |  | 1.000 | 0.181 | 0.671 | 0.671 |
| ApoB → log(PWRI) | 1.173 | 0.503 | 0.639 | 0.639 |  | 1.000 | 3.041 | 0.081 | 0.163 |
| log(PWRI) → HDL-C | 1.000 | 0.149 | 0.699 | 0.699 |  | 3.194 | 0.917 | 0.457 | 0.561 |
| HDL-C → log(PWRI) | 1.000 | 0.291 | 0.590 | 0.699 |  | 1.000 | 0.338 | 0.561 | 0.561 |
| log(PWRI → LDL-C | 4.141 | 1.611 | 0.205 | 0.410 |  | 1.000 | 0.002 | 0.963 | 0.963 |
| LDL-C → log(PWRI) | 1.000 | 0.149 | 0.699 | 0.699 |  | 1.000 | 4.036 | 0.045 | 0.089 |
| log(PWRI) → log(LpA) | 1.000 | 2.167 | 0.141 | 0.219 |  | 3.032 | 1.271 | 0.321 | 0.321 |
| log(LpA) → log(PWRI) | 7.018 | 1.261 | 0.219 | 0.219 |  | 1.714 | 2.200 | 0.107 | 0.215 |
| log(PWRI) → log(TG) | 1.000 | 0.113 | 0.737 | 0.834 |  | 1.711 | 0.669 | 0.522 | 0.522 |
| log(TG) → log(PWRI) | 1.000 | 0.044 | 0.834 | 0.834 |  | 1.527 | 2.141 | 0.087 | 0.174 |
| log(PWRI) → TC | 3.947 | 1.115 | 0.442 | 0.848 |  | 1.000 | 0.170 | 0.681 | 0.681 |
| TC → log(PWRI) | 1.000 | 0.037 | 0.848 | 0.848 |  | 1.000 | 4.982 | 0.026 | 0.052 |
| log(PWPP) → ApoA | 1.000 | 2.459 | 0.117 | 0.234 |  | 4.520 | 1.598 | 0.147 | 0.294 |
| ApoA → log(PWPP) | 1.000 | 0.272 | 0.602 | 0.602 |  | 1.949 | 0.870 | 0.457 | 0.457 |
| log(PWPP) → ApoB | 1.171 | 0.466 | 0.656 | 0.752 |  | 1.873 | 2.017 | 0.110 | 0.220 |
| ApoB → log(PWPP) | 1.000 | 0.100 | 0.752 | 0.752 |  | 2.632 | 1.425 | 0.230 | 0.230 |
| log(PWPP) → HDL-C | 1.803 | 0.930 | 0.471 | 0.637 |  | 4.128 | 1.274 | 0.267 | 0.534 |
| HDL-C → log(PWPP) | 1.000 | 0.223 | 0.637 | 0.637 |  | 1.389 | 0.248 | 0.815 | 0.815 |
| log(PWPP) → LDL-C | 1.361 | 0.470 | 0.703 | 0.703 |  | 3.447 | 2.170 | 0.062 | 0.108 |
| LDL-C → log(PWPP) | 1.000 | 0.157 | 0.692 | 0.703 |  | 1.000 | 2.580 | 0.108 | 0.108 |
| log(PWPP) → log(LpA) | 1.642 | 1.871 | 0.146 | 0.152 |  | 1.000 | 4.818 | 0.028 | 0.057 |
| log(LpA) → log(PWPP) | 1.000 | 2.056 | 0.152 | 0.152 |  | 1.000 | 0.236 | 0.627 | 0.627 |
| log(PWPP → log(TG) | 1.000 | 0.447 | 0.504 | 0.504 |  | 1.000 | 0.131 | 0.718 | 0.718 |
| log(TG) → log(PWPP) | 7.877 | 1.597 | 0.104 | 0.207 |  | 2.345 | 2.010 | 0.110 | 0.221 |
| log(PWPP) → TC | 1.000 | 0.581 | 0.446 | 0.601 |  | 4.111 | 1.961 | 0.079 | 0.158 |
| TC → log(PWPP) | 2.461 | 0.656 | 0.601 | 0.601 |  | 4.003 | 1.457 | 0.204 | 0.204 |
| PR → ApoA | 4.059 | 2.180 | 0.052 | 0.103 |  | 1.000 | 0.016 | 0.899 | 0.899 |
| ApoA → PR | 1.000 | 0.778 | 0.378 | 0.378 |  | 1.158 | 0.182 | 0.849 | 0.899 |
| PR → ApoB | 1.000 | 0.123 | 0.726 | 0.726 |  | 1.457 | 0.981 | 0.471 | 0.511 |
| ApoB → PR | 1.381 | 1.022 | 0.255 | 0.510 |  | 2.494 | 0.809 | 0.511 | 0.511 |
| PR → HDL-C | 3.764 | 2.516 | 0.028 | 0.057 |  | 1.133 | 0.159 | 0.858 | 0.858 |
| HDL-C → PR | 1.000 | 0.321 | 0.571 | 0.571 |  | 1.000 | 0.037 | 0.848 | 0.858 |
| PR → LDL-C | 1.000 | 0.516 | 0.473 | 0.473 |  | 7.506 | 1.924 | 0.047 | 0.094 |
| LDL-C → PR | 2.180 | 0.927 | 0.350 | 0.473 |  | 3.015 | 1.274 | 0.243 | 0.243 |
| PR → log(LpA) | 1.000 | 0.075 | 0.784 | 0.784 |  | 1.725 | 1.407 | 0.274 | 0.547 |
| log(LpA) → PR | 1.172 | 1.007 | 0.413 | 0.784 |  | 1.000 | 0.141 | 0.707 | 0.707 |
| PR → log(TG) | 1.000 | 1.755 | 0.185 | 0.371 |  | 3.861 | 2.148 | 0.060 | 0.060 |
| log(TG) → PR | 1.081 | 0.183 | 0.798 | 0.798 |  | 1.000 | 5.527 | 0.019 | 0.038 |
| PR → TC | 2.881 | 0.960 | 0.374 | 0.374 |  | 7.856 | 2.400 | 0.009 | 0.018 |
| TC → PR | 2.324 | 1.076 | 0.326 | 0.374 |  | 2.543 | 1.228 | 0.279 | 0.279 |
| Note: EDF indicates the effective degrees of freedom for the cross-lagged regression. Benjamini-Hochberg false discovery rate-corrected (FDR) P-value for multiple testing. Analyses were adjusted for Age, Smoking, Diabetes, Glucose, eGFR, CRP, IGF1, BMI, Albumin, ALP, ALT, AST, GGT, Phosphate, TBIL, TP, CysC, Urea, Urate, Testosterone, SHBG, Calcium, and VitD at baseline.  Abbreviations: Abbreviations: ASI, arterial stiffness index; PWRI, pulse wave reflection index; PWPP, pulse wave peak to peak time; PR, pulse rate; TC, cholesterol; HDL-C, high-density lipoprotein cholesterol; LDL-C, low-density lipoprotein cholesterol; TG, triglycerides; LpA, lipoprotein A; ApoA, apolipoprotein A; ApoB, apolipoprotein B; | | | | | | | | | |
